# Supplementary material for: Base-Resolution Methylome of Retinal Pigment Epithelial Cells Used in the First Trial of Human Induced Pluripotent Stem Cell-Based Autologous Transplantation
Source: Stem Cell Reports. 2019 Sep 26;13(4):761–74. doi: 10.1016/j.stemcr.2019.08.014 (PMC6829753; doi:10.1016/j.stemcr.2019.08.014)
Supplement: Document S1. Supplemental Experimental Procedures and Figures S1–S6 [file mmc1.pdf]

Stem Cell Reports, Volume *13*

Supplemental Information

Base-Resolution Methylome of Retinal Pigment Epithelial Cells Used in the First Trial of Human Induced Pluripotent Stem Cell-Based Autologous Transplantation

Hiromitsu Araki, Fumihito Miura, Akira Watanabe, Chikako Morinaga, Fumiyo Kitaoka, Yuko Kitano, Noriko Sakai, Yumiko Shibata, Motoki Terada, So Goto, Shinya Yamanaka, Masayo Takahashi, and Takashi Ito

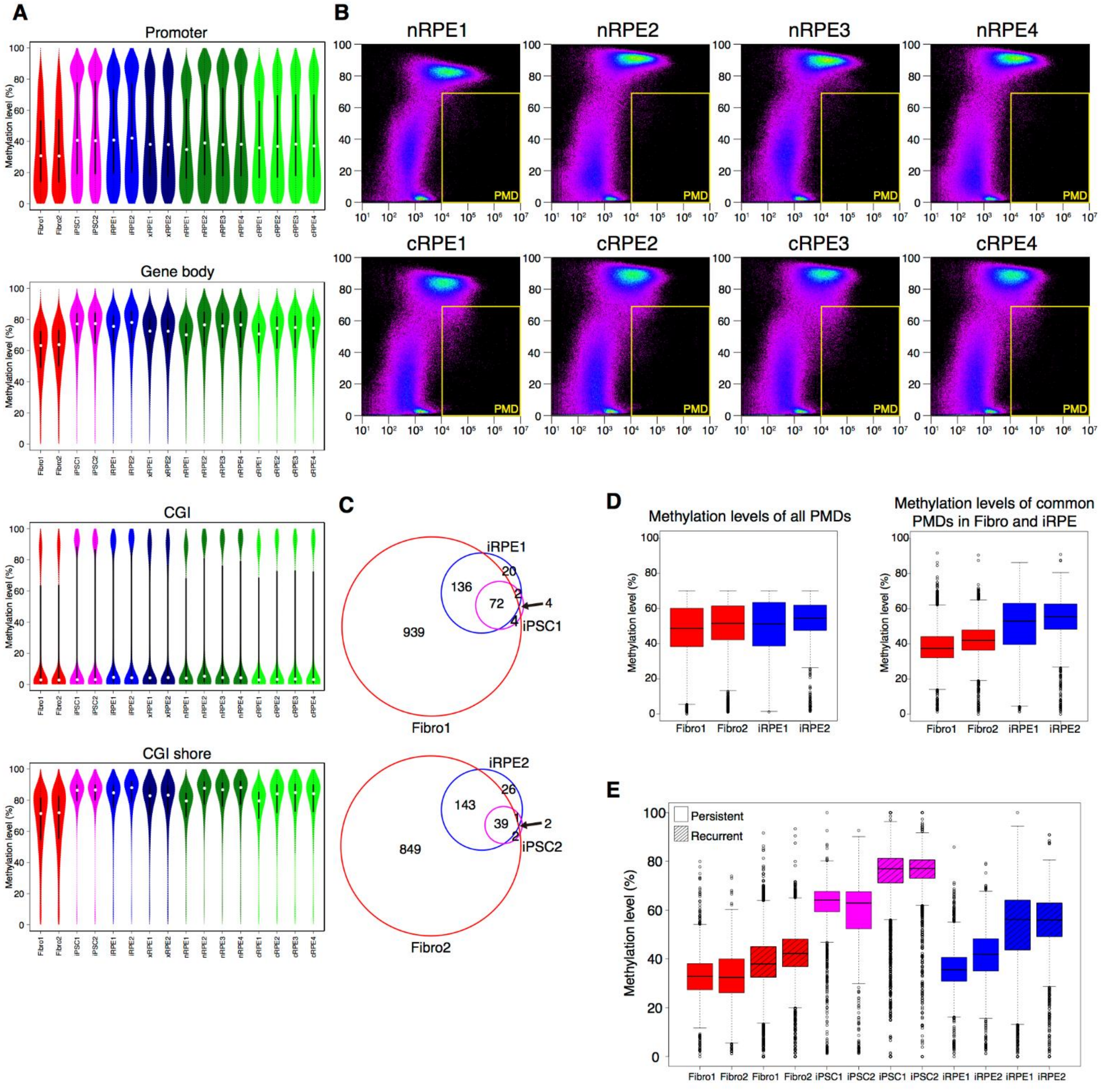

Figure S1. Characterization of PMDs. Related to Figure 1.

(A) Violin plots of mCG levels for indicated genomic features. Promoter, genomic region spanning 2 kb upstream of TSS; CGI shore, genomic region spanning 2 kb upstream and downstream of each CGI.

(B) MDL plots for all nRPE and cRPE samples.

(C) Venn diagram for overlap among PMDs in fibroblasts, iPSCs, and iRPE. The number indicates the total size (Mb) of genomic regions included in each section.

(D) Box plots for mCG levels of all and common PMDs in fibroblasts and iRPE.

(E) Box plots for mCG levels of persistent and recurrent PMDs in fibroblasts, iPSCs, and iRPE.

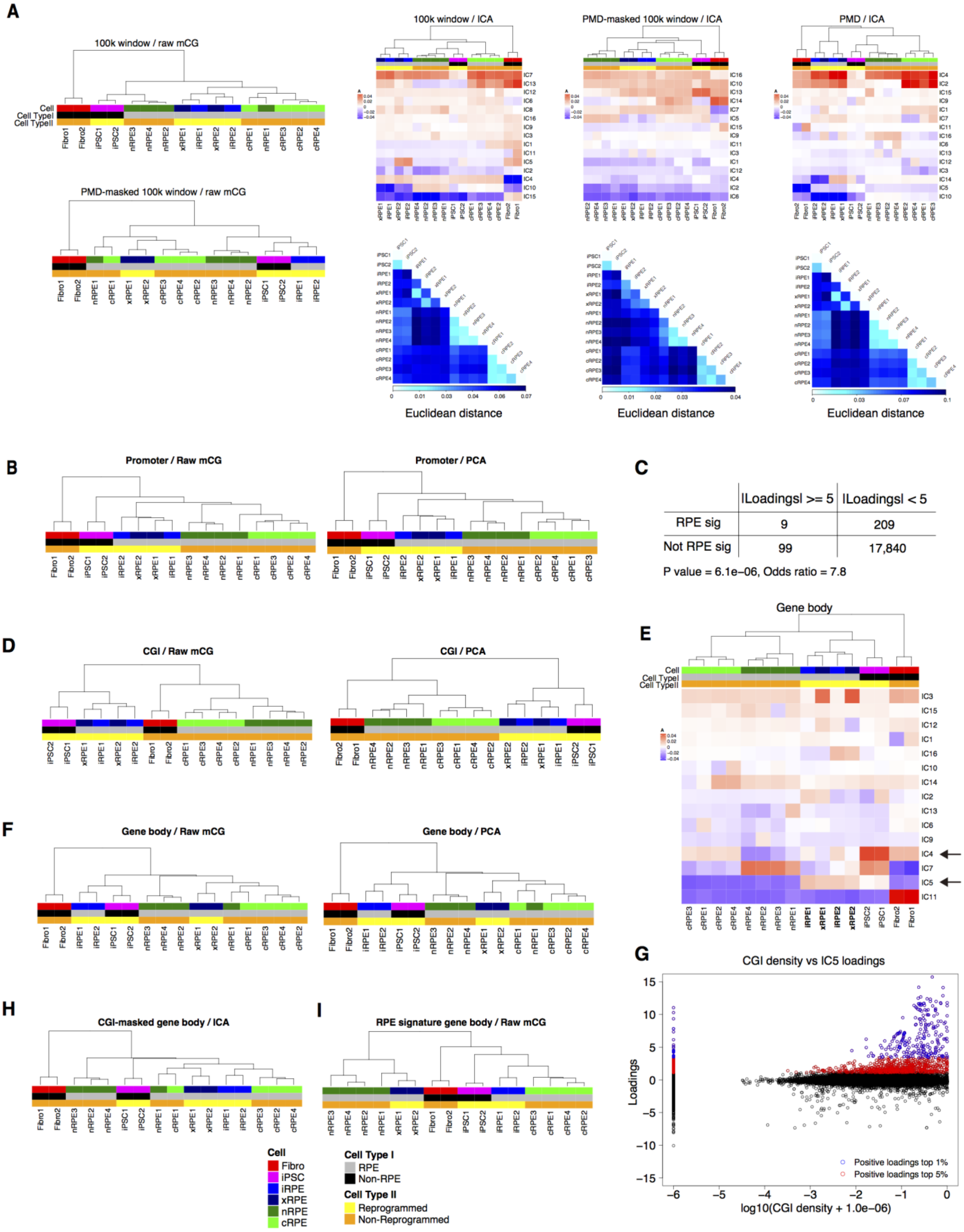

C

	|Loadings| >= 5	|Loadings| < 5
RPE sig	9	209
Not RPE sig	99	17,840

P value = 6.1e−06, Odds ratio = 7.8

Figure S2. Genomic Feature-Dependent Differential Clustering. Related to Figure 2.

(A) Clustering using 100-kb sliding windows. Results of raw mCG level-based clustering are shown for the whole and PMD-masked genomes (left). Results of ICA-based clustering are shown for the whole genome, PMD-masked genome, and PMDs with color-coded Euclidean distance matrices calculated from the columns of the mixing matrix A of ICA (right). Genomic regions judged as PMDs in fibroblasts are used for masking.

(B) Raw mCG level-based and PCA-based clustering using promoters.

(C) Enrichment of RPE-signature genes in promoters with large contributions to IC12 (absolute value of loadings ≥ 5).

(D) Raw mCG level-based and PCA-based clustering using CGIs.

(E) ICA-based clustering using gene bodies. Arrows indicate the rows corresponding to IC4 and IC5, which show differential weighting between cells in vitro and those in vivo and between reprogrammed and non-reprogrammed cells, respectively.

(F) Raw mCG level-based and PCA-based clustering using gene bodies.

(G) CGI density and factor loadings for IC5 of clustering using gene bodies. CGI density represents the ratio of total nucleotides in CGIs in a gene body to those of the gene body.

(H) ICA-based clustering using CGI-masked gene bodies.

(I) Raw mCG level-based clustering using RPE-signature gene bodies.

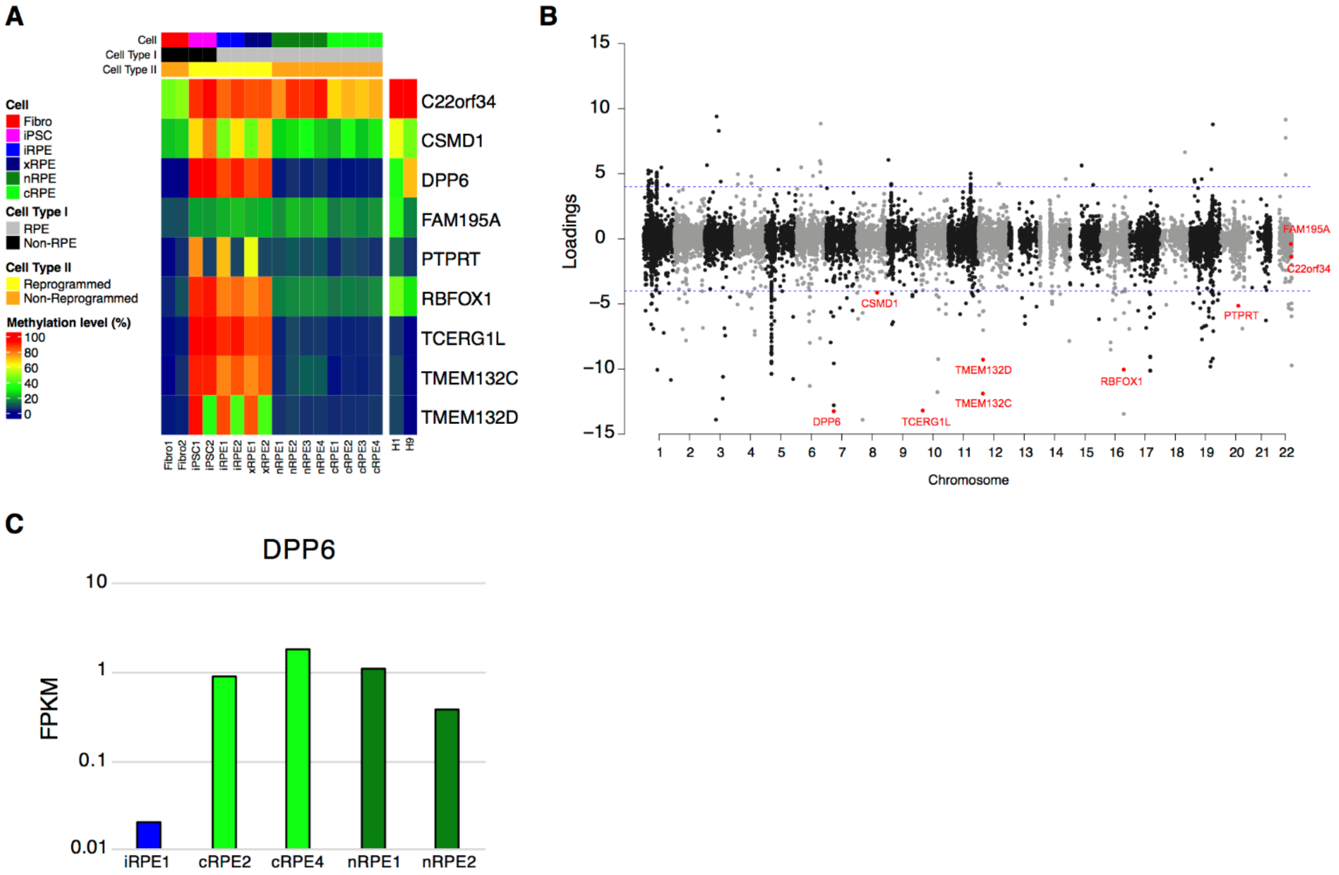

Figure S3. Reprogramming-associated Epigenetic Signatures. Related to Figure 4.

(A) Methylation heat map of reprogramming-associated epigenetic signature genes. Colors indicate the methylation levels of promoters.

(B) Manhattan plot of loadings for IC5 of ICA-based clustering of promoters. Reprogramming-associated epigenetic signature genes are indicated in red. The blue dashed lines indicate the 99th percentiles for the absolute values of loadings.

(C) Expression levels of *DPP6* in iRPE1, cRPE2/4, and nRPE1/2**.**

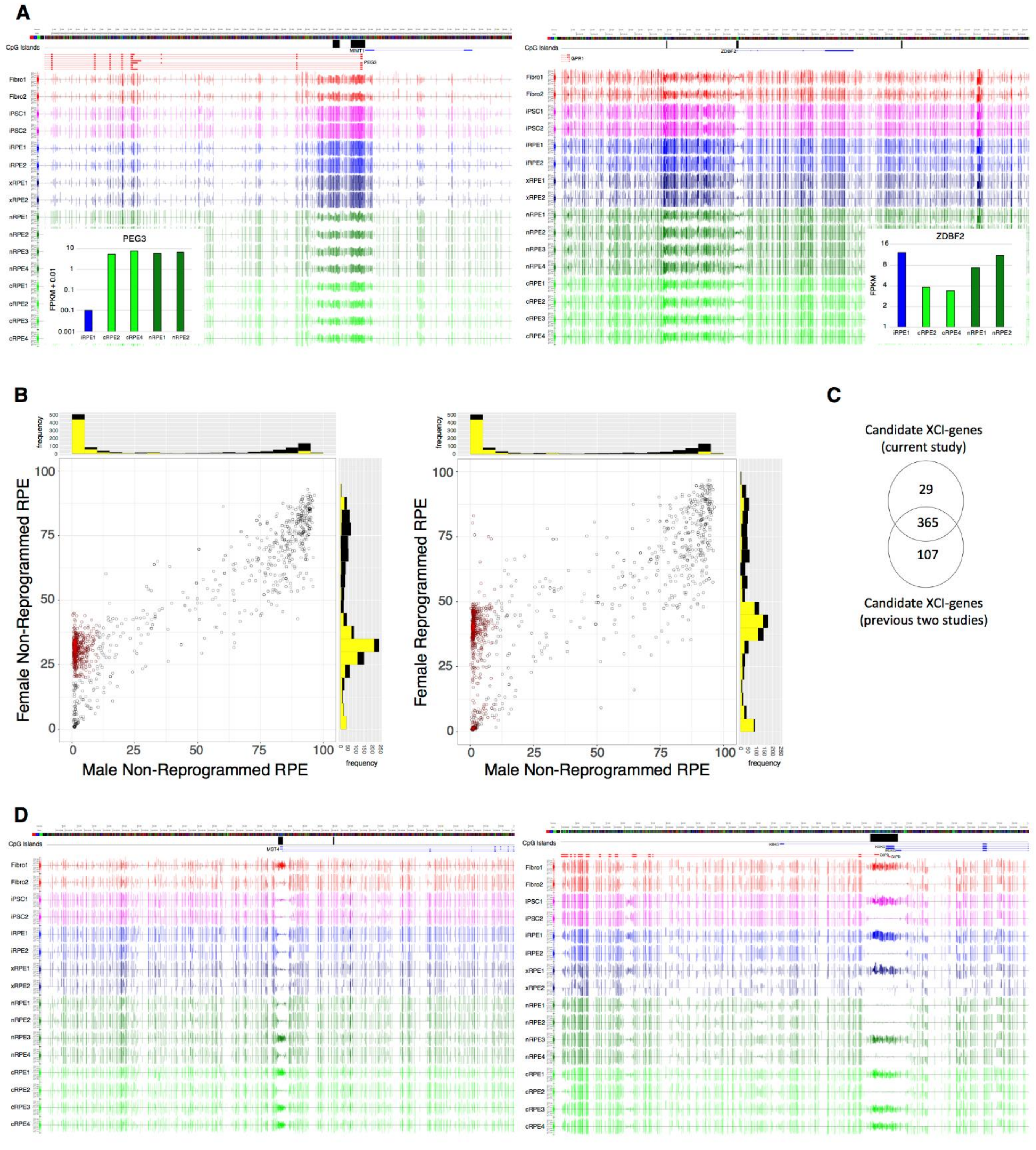

Figure S4. Loss of Imprinting and Erosion of XCI. Related to Figure 4.

(A) Genome browser shots for imprinted genes *PEG3* and *ZDBF2*. From top to bottom, tracks represent CGIs, gene models, and mCG levels of both strands in Fibro1, 2; iPSC1, 2; iRPE1, 2; xRPE1, 2; nRPE1–4; and cRPE1–4. Insets indicate their expression levels.

(B) Identification of candidate XCI-genes. A gene is regarded as a candidate XCI-gene (red circle), if the methylation level of its TSS-proximal region is ≤10% and 20–45% in male and female non-reprogrammed cells, respectively (left panel). Methylation levels of TSS-proximal regions of X-linked genes are also compared between the reprogrammed and non-reprogrammed female RPE cells (right). The yellow portions of the histograms along X- and Y-axes indicate TSS-proximal regions overlapping with CGIs.

(C) Overlap between candidate XCI-genes identified in this study and previous studies (Balaton et al., 2015; Tukiainen et al. 2017).

(D) Genome browser shot for *MST4* and *G6PD*. Methylation patterns of promoter CGIs indicate that XCI appears to be lost and maintained for *MST4* and *G6PD*, respectively.

A

Genes		Down-regulation in iRPE	
		+	–
Proximal hyper-DMRs	+	59	1,195
	–	244	16,543

P value = 3.7e–13, Odds ratio = 3.3

Genes		Up-regulation in iRPE	
		+	–
Proximal hypo-DMRs	+	13	192
	–	131	17,705

P value = 1.0e–08, Odds ratio = 9.1

B

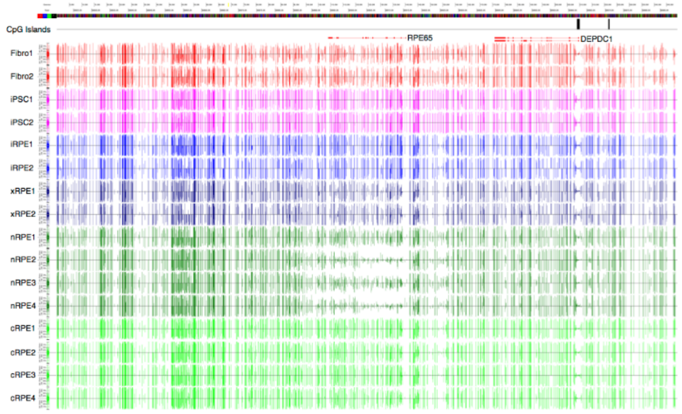

C

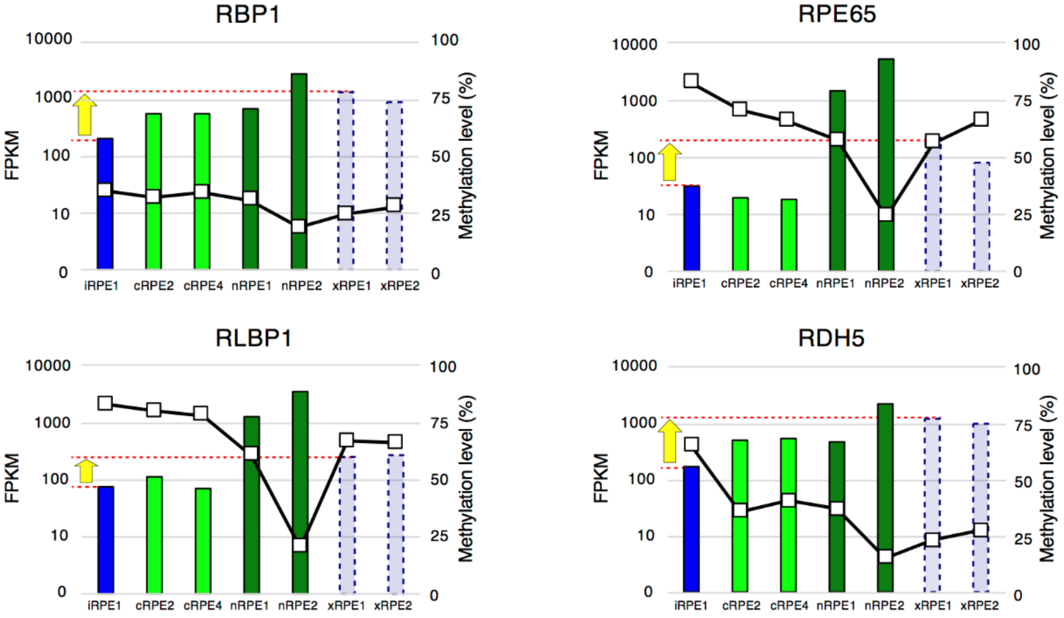

D

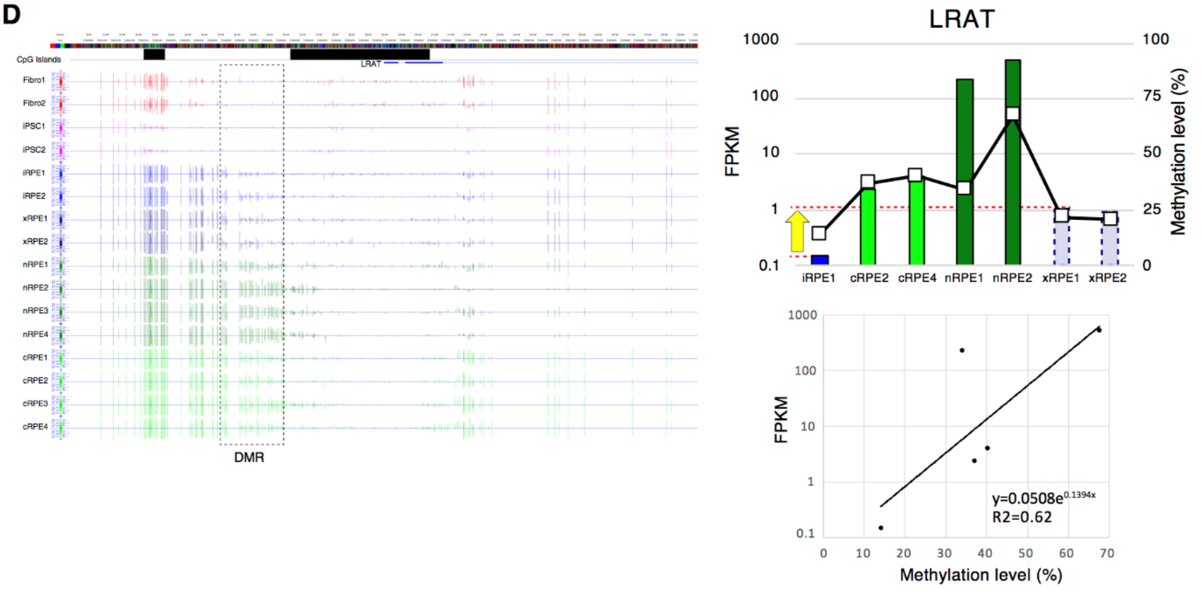

Figure S5. Differential Methylation and Gene Expression. Related to Figure 5.

(A) Correlation between DMRs and DEGs. Co-occurrence is significant between genes proximal to hyper-DMRs and genes down-regulated in iRPE1 (top) and between genes proximal to hypo-DMRs and genes up-regulated in iRPE relative to nRPE (bottom).

(B) Genome browser shot for *RPE65*. From top to bottom, tracks represent CGIs, gene models, and mCG levels of both strands in Fibro1, 2; iPSC1, 2; iRPE1, 2; xRPE1, 2; nRPE1–4; and cRPE1–4. Note that gene body of *RPE65* is hypomethylated in nRPE1–4 compared to the other cells.

(C) Expression levels of 4 visual-cycle genes in xRPE estimated from their gene-body methylation levels. Bars indicate expression levels, and line charts indicate gene-body methylation levels. Bars with dotted lines indicate the estimated expression levels in xRPE. Yellow arrows indicate the estimated levels of induction of individual genes after xenotransplantation.

(D) Differential methylation and expression of *LRAT*. DMR between iRPE and nRPE is bracketed in the genome browser shot (left). Expression levels of *LRAT* in xRPE are estimated from the methylation level of DMR (right top) and the correlation between the methylation level of DMR and expression level of *LRAT* mRNA (right bottom). Yellow arrow indicates the estimated induction of *LRAT* after xenotransplantation.

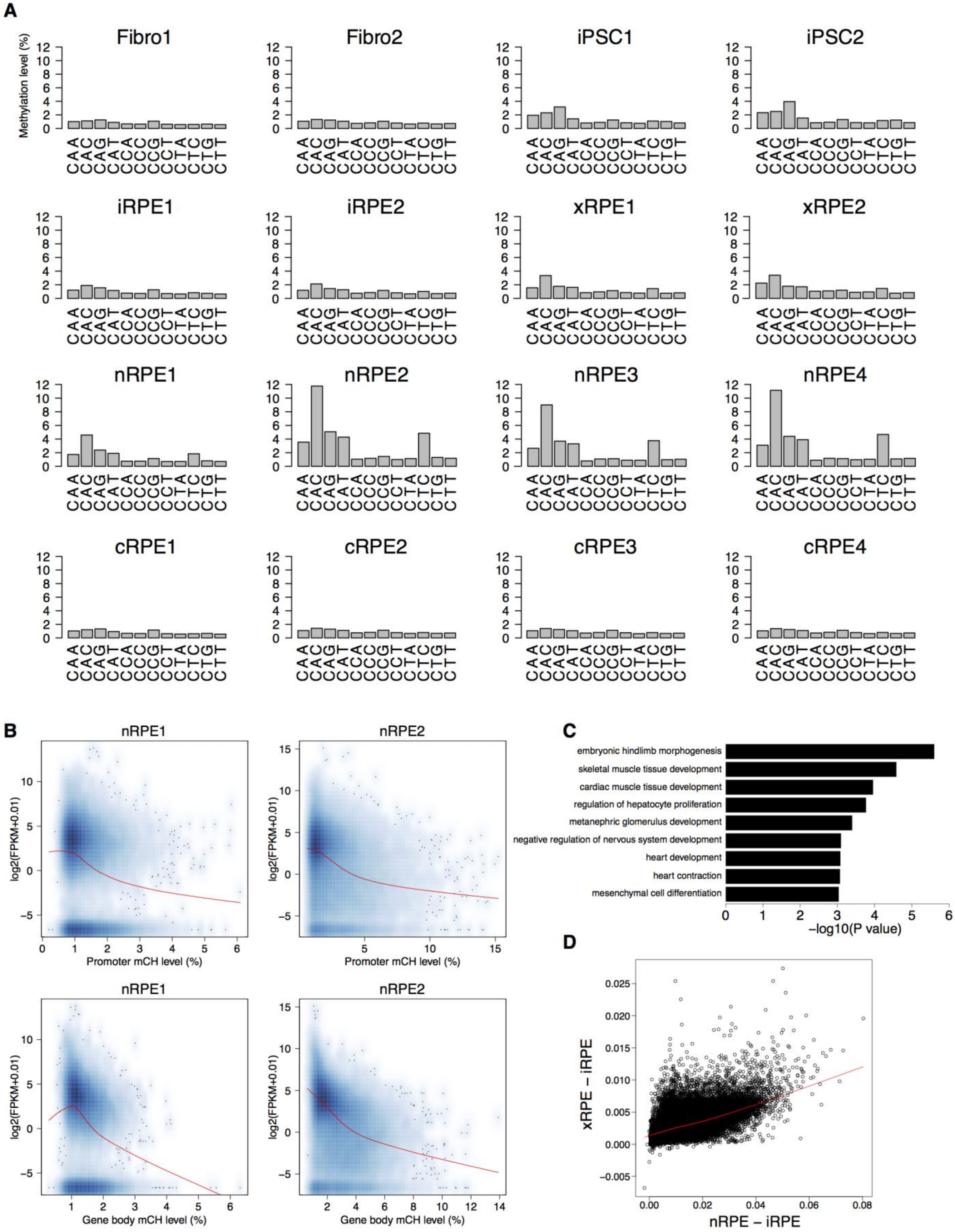

A
Fibro1
Fibro2
iPSC1
iPSC2
iRPE1
iRPE2
xRPE1
xRPE2
nRPE1
nRPE2
nRPE3
nRPE4
cRPE1
cRPE2
cRPE3
cRPE4
Methylation level (%)
CAA CAC CAG CAT CCA CCC CCG CCT CTA CTC CTG CTT
B
nRPE1
nRPE2
log2(FPKM+0.01)
Promoter mCH level (%)
Gene body mCH level (%)
C
embryonic hindlimb morphogenesis
skeletal muscle tissue development
cardiac muscle tissue development
regulation of hepatocyte proliferation
metanephric glomerulus development
negative regulation of nervous system development
heart development
heart contraction
mesenchymal cell differentiation
−log10(P value)
D
xRPE − iRPE
nRPE − iRPE

Figure S6. Characterization of Non-CpG Methylation. Related to Figure 6.

(A) Sequence contexts and mCH levels in each of the 16 methylomes.

(B) Correlation between the levels of mCHs and mRNAs in nRPE1 and nRPE2.

(C) GO enrichment analysis of top 500 genes with high promoter mCH levels in nRPE.

(D) Correlation of mCHs between nRPE and xRPE. Each circle indicates a 100-kb window. For each circle, X- and Y-coordinates indicate the difference of mCH levels (Δ%mCH) between nRPE and iRPE and the Δ%mCH between xRPE and iRPE, respectively.

Supplemental Experimental Procedures

Sample preparation

cRPE: We obtained four distinct batches of normal human retinal pigment cells from Lonza (Lonza, 00194987) and used them as cRPE samples. These cells were cryopreserved, primary cells that were packaged at passage 2. They were guaranteed through 5 population doublings and stain positive (≥ 90%) for pancytokeratin and Zo-1 and negative (≤ 10%) for fibroblast contamination and endothelial marker CD31 (≤ 1%). We spread them on dishes coated with CELLstart (Thermo Fisher). Following cultivation in RtEGM™ BulletKit™ (Lonza, 00195409) supplemented with 2% FBS for 2 to 3 days, we switched the medium to RPE maintenance medium [DMEM low glucose (Sigma-Aldrich, D6046) 350mL, F12 (Sigma-Aldrich, N6658) 150mL, B27 (Thermo Fisher, 17504) 10mL, L-glutamine solution (Sigma-Aldrich, G7513) 5mL, Penicillin-Streptomycin solution (Thermos Fisher, 15140) 5mL] supplemented with 10 ng/mL bFGF (Wako) and 0.5 µM SB431542 (Sigma-Aldrich, S4317).

nRPE: We prepared four nRPE samples from eyes of four healthy donors obtained from SightLife TM (http://www.sightlife.org/, Seattle, WA). We used the SRIRS (simultaneous RPE isolation and RNA stabilization) method based on a unique feature of RNAprotect Cell Reagent (Qiagen 76526) to detaches RPE cells from surgically prepared posterior eye cup, thus providing a highly purified RPE preparation (Wang et al., 2012).

xRPE: We prepared two xRPE samples as described previously (Mandai et al., 2017). Briefly, 1×10^6 iRPE cells were subcutaneously injected with Matrigel in NOG mice. The samples analyzed in this study, xRPE1 and xRPE2, were recovered from the mice at 25 and 9 weeks after transplantation, respectively. Histological examination of these xenografts failed to find any abnormality.

SNP-masked patients' methylome data

We prepared methylation level data on all C's in the reference genome, except for those serving as SNP sites with minor allele frequency > 0.01 in Japanese population as follows. Based on SNP frequency data from TogoVar, an integrated database for Japanese genome variants/variations (https://togovar.biosciencedbc.jp/), we selected 4,558,776 C's from both strands of the reference genome (i.e., 0.39% of total C's) and masked them from the methylation level data for the prevention of potential personal identification. The data include the numbers of reads supporting methylated and unmethylated status of each C.

RNA-seq

RNA-Seq libraries were prepared and sequenced as described previously (Mandai et al., 2017). RNA-Seq reads were mapped to the hg19 reference genome using Tophat2. Cufflinks (Trapnell et al., 2010) was used to generate the normalized expression levels of genes expressed as FPKM values.

Mapped reads were assigned to all exons using featureCounts (Liao et al., 2014). To filter out genes with low expression levels, we considered only those with at least 10 read counts in either iRPE1 or nRPE1/2. The differential analysis was conducted using the Bioconductor package edgeR (Robinson et al., 2009), applying TMM (trimmed Mean of M-values) library normalization. A gene was defined as differentially expressed, if the false discovery rate (FDR)

corrected with Benjamini-Hochberg method was less than 0.01 and if the log2 fold change (log2FC) was more than 1 (up-regulated) or less than −1 (down-regulated).

Software and algorithm

Versions and sources of the softwares/algorithms used in this study are summarized in the following table.

REAGENT or RESOURCE	IDENTIFIER	SOURCE
ComplexHeatmap (v1.12.0)	Gu et al., 2016	https://bioconductor.org/packages/release/bioc/html/ComplexHeatmap.html
edgeR (v3.16.5)	Robinson et al., 2010	https://bioconductor.org/packages/release/bioc/html/edgeR.html
fastICA (v1.2-1)	Marchini et al., 2017	https://cran.r-project.org/web/packages/fastICA/index.html
featureCounts (v1.4.6)	Liao et al., 2014	http://bioinf.wehi.edu.au/featureCounts/
HOMER (v4.9.1)	Heinz et al., 2010	http://homer.ucsd.edu/homer/index.html
metilene (v0.2-6)	Jühling et al., 2016	https://www.bioinf.uni-leipzig.de/Software/metilene/
R (v3.3.3)	R Core Team	http://www.R-project.org/
topGO (v2.26.0)	Alexa and Rahnenfuhrer, 2016	http://bioconductor.org/packages/release/bioc/html/topGO.html

Supplemental References

Balaton, B.P., Cotton, A.M., and Brown, C.J. (2015). Derivation of consensus inactivation status for X-linked genes from genome-wide studies. Biol. Sex Differ. 6, 35.

Gu, Z., Eils, R., and Schlesner, M. (2016). Complex heatmaps reveal patterns and correlations in multidimensional genomic data. Bioinformatics 32, 2847–2849.

Heinz, S., Benner, C., Spann, N., Bertolino, E., Lin, Y.C., Laslo, P., Cheng, J.X., Murre, C., Singh, H., and Glass, C.K. (2010). Simple combinations of lineage-determining transcription factors prime cis-regulatory elements required for macrophage and B cell identities. Mol. Cell 38, 576–589.

Jühling, F., Kretzmer, H., Bernhart, S.H., Otto, C., Stadler, P.F., and Hoffmann, S. (2016). metilene: fast and sensitive calling of differentially methylated regions from bisulfite sequencing data. Genome Res. 26, 256–262.

Liao, Y., Smyth, G.K., and Shi, W. (2014). featureCounts: an efficient general purpose program for assigning sequence reads to genomic features. Bioinformatics 30, 923–930.

Mandai, M., Watanabe, A., Kurimoto, Y., Hirami, Y., Morinaga, C., Daimon, T., Fujihara, M., Akimaru, H., Sakai, N., Shibata, Y., et al. (2017). Autologous induced stem-cell-derived retinal cells for macular degeneration. N. Engl. J. Med. 376, 1038–1046.

Robinson, M.D., McCarthy, D.J., and Smyth, G.K. (2010). edgeR: a Bioconductor package for differential expression analysis of digital gene expression data. Bioinformatics 26, 139–140.

Trapnell, C., Williams, B.A., Pertea, G., Mortazavi, A., Kwan, G., van Baren, M.J., Salzberg, S.L., Wold, B.J., and Pachter, L. (2010). Transcript assembly and quantification by RNA-seq reveals unannotated transcripts and isoform switching during cell differentiation. Nat. Biotechnol. 28, 511–515.

Tukiainen, T., Villani, A.C., Yen, A., Rivas, M.A., Marshall, J.L., Satija, R., Aguirre, M., Gauthier, L., Fleharty, M., Kirby, A. et al. (2017). Landscape of X chromosome inactivation across human tissues. Nature 550, 244–248.

Wang, C.X-Z., Zhang, K., Aredo, B., Lu, H., and Ufret-Vincenty, R.L. (2012) Novel method for the rapid isolation of RPE cells specifically for RNA extraction and analysis. Exp. Eye Res. 102, 1–9.
