# Supplementary material for: Base-Resolution Methylome of Retinal Pigment Epithelial Cells Used in the First Trial of Human Induced Pluripotent Stem Cell-Based Autologous Transplantation
Source: Stem Cell Reports. 2019 Sep 26;13(4):761–74. doi: 10.1016/j.stemcr.2019.08.014 (PMC6829753; doi:10.1016/j.stemcr.2019.08.014)
Supplement: Document S2. Article plus Supplemental Information [file mmc3.pdf]

Stem Cell Reports

ISSCR

Resource

OPEN ACCESS

Base-Resolution Methylome of Retinal Pigment Epithelial Cells Used in the First Trial of Human Induced Pluripotent Stem Cell-Based Autologous Transplantation

Hiromitsu Araki,[1] Fumihito Miura,[1] Akira Watanabe,[2] Chikako Morinaga,[3] Fumiyo Kitaoka,[2] Yuko Kitano,[2] Noriko Sakai,[3] Yumiko Shibata,[3] Motoki Terada,[3] So Goto,[3] Shinya Yamanaka,[2] Masayo Takahashi,[3] and Takashi Ito[1,*]

[1]Department of Biochemistry, Kyushu University Graduate School of Medical Sciences, Fukuoka 812-8582, Japan
[2]Center for iPS Cell Research and Application, Kyoto University, Kyoto 606-8507, Japan
[3]Laboratory for Retinal Regeneration, Center for Biosystems Dynamics Research, RIKEN, Kobe 650-0047, Japan
*Correspondence: tito@med.kyushu-u.ac.jp
https://doi.org/10.1016/j.stemcr.2019.08.014

SUMMARY

The first-in-human trial of induced pluripotent stem cell (iPSC)-based autologous transplantation was successfully performed on a female patient with age-related macular degeneration. Here we delineated the base-resolution methylome of the iPSC-derived retinal pigment epithelium (iRPE) used in this trial. The methylome of iRPE closely resembled that of native RPE (nRPE), although partially methylated domains (PMDs) emerged in iRPE but not nRPE. Most differentially methylated regions between iRPE and nRPE appeared to originate from (de)methylation errors during differentiation, whereas errors at reprogramming resulted in aberrant genomic imprinting and X chromosome reactivation. Moreover, non-CpG methylation was prominent in nRPE but not iRPE. Intriguingly, xenotransplantation to mouse remodeled the iRPE methylome to demethylate a subset of suppressed genes and accumulate non-CpG methylation, but failed to resolve PMDs and hypermethylated CpG islands. Although the impacts of these alterations remain elusive, our findings should provide a useful guide for methylome analyses of other iPSC-derived cells.

INTRODUCTION

Induced pluripotent stem cell (iPSC) technology is rapidly revolutionizing diverse fields in biomedical sciences (Inoue et al., 2014; Tapia and Schöler, 2016; Trounson and DeWitt, 2016). Notably, patient-derived iPSCs provide an invaluable source for deriving the affected cells in a specific patient, which can serve as an optimal model for the patient's disease. Accordingly, disease modeling using patient-derived iPSC has been overcoming various limitations inherent to other models, and this has been markedly accelerating both the mechanistic understanding of pathogenesis and the chemical screening for potential therapeutics. As well as being employed in basic investigations, cells differentiated from patient-derived iPSCs can also be used to predict drug response and side effects for stratifying patients in the clinic. Furthermore, patient-derived iPSCs have an unsurpassed impact on cell transplantation because they can serve as a source for deriving autografts that should have minimal risk of rejection and other adverse immune responses. The recent advent of genome-editing technologies has even enabled the correction of genetic insults *ex vivo* before autologous transplantation, which has further enhanced the power of regenerative medicine.

The first-in-human trial of iPSC-derived autologous transplantation was performed on a female patient with exudative age-related macular degeneration (AMD) (Mandai et al., 2017). AMD is a leading cause of visual impairment in elderly people and involves progressive degeneration of the retinal pigment epithelium (RPE), which plays a critical role in photoreceptor maintenance. The RPE represents an optimal tissue for replacement therapy performed using an iPSC-derived autologous graft because RPE cells are exceptionally safe, terminally differentiated cells that have not been found to undergo malignant transformation in any study reported thus far. Furthermore, a robust protocol has been established to prepare human iPSC-derived RPE sheets that meet clinical-use requirements in terms of quality, quantity, consistency, and safety (Kamao et al., 2014). In the first trial, iPSCs were generated from the patient's skin fibroblasts and used to derive the RPE, and a sheet of the iPSC-derived RPE (iRPE) was transplanted into a subretinal region of the patient's eye. The sheet survived well without showing any signs of rejection or unexpected proliferation at 1 year after surgery, thus successfully retaining the initial goal of the trial as reported previously (Mandai et al., 2017). At the time of preparation of this manuscript, the sheet has been properly functioning for 4 years, without causing any clinical problems in the patient (unpublished data).

Before transplantation, the iRPE sheet was subjected to a series of rigorous examinations, which included tests for tumorigenicity, genome integrity, and various functionalities. DNA methylation was also examined because the reprogramming of methylation is frequently incomplete,

This is an open access article under the CC BY license (http://creativecommons.org/licenses/by/4.0/).

and the aberrant status can be transmitted to iPSC-derived cells (Huo et al., 2014; Liang and Zhang, 2013). These aberrations, which include hypermethylation of CpG islands (CGIs), loss of genomic imprinting, and incomplete inactivation of female X chromosome, could be transmitted to the iRPE and thus pose a potential risk of abnormal proliferation and compromised functions. Therefore, a targeted methylome analysis, using the well-established Infinium 450K methylation array, was conducted to confirm that the iRPE methylome was not only closer to that of the RPE than those of other tissues, but also free from apparent risks such as the CGI methylation of tumor-suppressor genes (Mandai et al., 2017).

To elucidate the iRPE methylome more comprehensively than before, we conducted whole-genome bisulfite sequencing (WGBS) of (1) fibroblasts from the female patient who received the iRPE transplantation (patient 1); (2) the iPSCs induced from the fibroblasts; (3) the iRPE sheet derived from the iPSCs; and (4) the iRPE xenotransplanted to the hypodermis of non-obese diabetic/SCID mice (xRPE) (Mandai et al., 2017). Moreover, we analyzed the same set of samples derived from a male patient (patient 2), transplantation to whom was not performed because of genetic insults identified in the genome of the patient's iRPE sheet and the relatively stable clinical symptoms of the patient (Mandai et al., 2017). We also examined four distinct batches of RPE cultured *in vitro* (cRPE) and native RPE obtained from four autopsy specimens (nRPE) (Mandai et al., 2017). A series of comparative analyses of these WGBS data revealed an overall resemblance and several differences between the iRPE and nRPE methylomes. Although a previous analysis performed using reduced representation bisulfite sequencing (RRBS) has been reported on human embryonic stem cell (ESC)-derived RPE, iPSC-derived RPE, and fetal RPE cell lines (Liu et al., 2014), our WGBS study is considerably more comprehensive and uncovers heretofore undescribed methylomic features of iRPE and nRPE.

RESULTS

Emergence of Partially Methylated Domains in iRPE

We used the post-bisulfite adaptor tagging (PBAT) method (Miura et al., 2012; Miura and Ito, 2018) to generate PCR-free WGBS data from the fibroblasts, iPSCs, iRPE, and xRPE derived from the two AMD patients reported previously (Mandai et al., 2017). Moreover, we similarly analyzed four each of the cRPE and nRPE samples as the controls for iRPE (Table S1).

The overall CpG methylation (mCG) levels were largely comparable among the 16 samples examined, except in the case of fibroblasts (Figures 1A and S1A). The low mCG levels detected in the fibroblasts were expected because these cells are known to harbor partially methylated domains (PMDs). PMDs were first detected in the human fibroblast cell line IMR90 as genomic regions that were larger than 10 kb in size and below 70% in mean mCG level, often overlapping with lamina-associated domains (LADs) (Lister et al., 2009). PMDs are also prominent in the placenta and in various cancer cells (Lorincz and Schübeler, 2017).

To screen for the presence of PMDs and other large methylomic alterations, we used a change-point detection algorithm to segment each methylome into distinct domains according to their mean mCG levels (Yokoyama et al., 2015). To visualize global trends in methylome segmentation, we displayed the domain composition of each methylome using a plot termed Methylated Domain Landscape (MDL), in which the mean mCG level of each domain was plotted against its size (Yokoyama et al., 2015) (Figures 1B and S1B). MDL plots confirmed that the patient-derived fibroblasts harbored prominent PMDs (Figure 1B). In agreement with the study on IMR90 cells (Lister et al., 2009), PMDs occupied up to approximately one-third of the patient-derived fibroblast genome (1,151 and 1,033 Mb in patients 1 and 2, respectively) (Figure 1C), and most of these PMDs (93% and 96% in patients 1 and 2) disappeared upon reprogramming, as also reported previously (Lister et al., 2011) (Figures 1B and S1C).

Notably, PMDs emerged during the differentiation from iPSCs to iRPE (Figures 1B and S1C). Compared with PMDs in fibroblasts, PMDs appeared in iRPE occupied considerably smaller regions in the genome (Figures 1B and 1C) and were less demethylated (Wilcoxon test, $p < 2.2 \times 10^{-16}$ in both patients 1 and 2) (Figure S1D). Importantly, most PMDs in iRPE (90% and 87% in patients 1 and 2, respectively) overlapped with PMDs in the fibroblasts, suggestive of their physiological nature (Figure 1C). The PMDs shared between fibroblasts and iRPE were less methylated in the fibroblasts than in the iRPE (Figure S1D), and their methylation levels were below the overall average in fibroblasts (Figure S1D). These common PMDs either recurred during differentiation (recurrent PMDs, 65% and 79% in patients 1 and 2, respectively) or inherited from iPSC (persistent PMDs, 35% and 21% in patients 1 and 2, respectively) (Figure S1C). As might be expected, methylation levels were generally lower in the persistent PMDs than in the recurrent PMDs (Figure S1E).

Intriguingly, in contrast to fibroblasts and iRPE, the nRPE harbored only a negligible level of PMDs (Figures 1B and S1B). Thus, iRPE failed to recapitulate the PMD paucity observed in nRPE. However, cRPE also displayed signs of PMD emergence, presumably as a physiological response to *in vitro* cultivation (Figures 1B and S1B). To

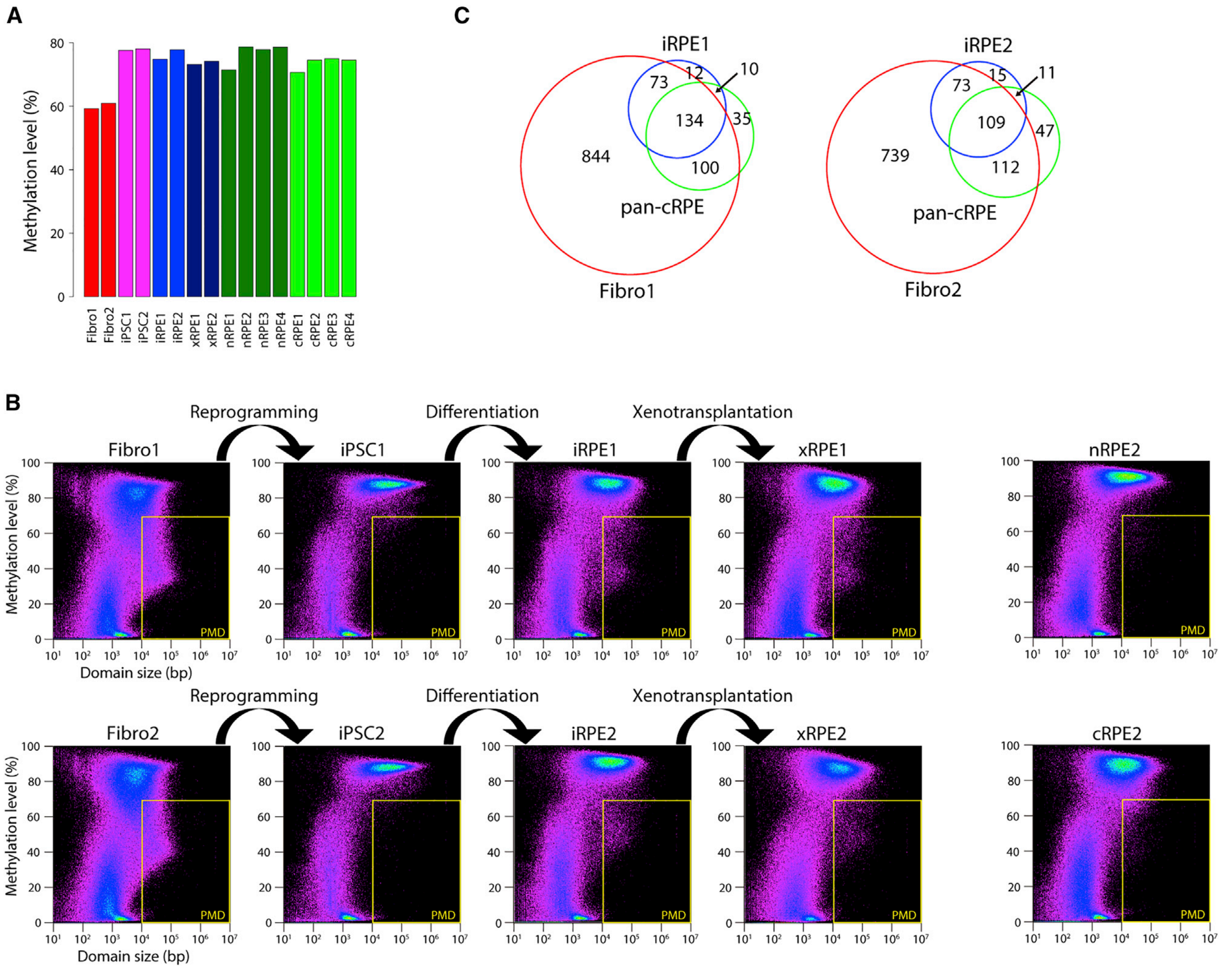

Figure 1. Emergence of PMDs in iRPE

(A) Mean mCG levels of the 16 methylomes determined in this study.

(B) MDL plots for methylome segmentation. For each domain defined by a change-point detection approach, mean methylation level in percentile (y axis) is plotted against log10 of its size in base pairs (x axis) (Yokoyama et al., 2015). Yellow lines demarcate domains that fulfill the original criteria of PMDs (size >10 kb; mean methylation level <70%) (Lister et al., 2009).

(C) Venn diagram for overlap among PMDs in fibroblasts, iRPE, and cRPE. Note that pan-cRPE represents the union of PMDs in cRPE1–4. The number indicates the total size (Mb) of genomic regions in each section of the Venn diagram.

approximate the physiological PMDs induced in normal RPE by *in vitro* cultivation, we assessed the combined PMDs in the four cRPE samples (pan-cRPE PMDs). Based on considering the pan-cRPE PMDs, we estimated that the iRPE-specific PMDs accounted for <5.2% and <7.2% of the PMDs in iRPE1 and iRPE2, respectively (Figure 1C). Therefore, induction of most if not all PMDs in iRPE was likely a physiological response to *in vitro* cultivation. Nevertheless, once induced, the PMDs appeared to be stable: they persisted even 1 year after xenotransplantation to mouse (i.e., they were retained in xRPE) (Figure 1B).

Methylomic Evidence for Proper Differentiation and Memory of Reprogramming in iRPE

To evaluate the overall resemblance among the 16 methylomes, we employed three hierarchical clustering methods, in each of which we used the raw mCG levels, the outputs of principal-component analysis (PCA), or those of independent component analysis (ICA).

We initially performed the clustering using the mean mCG levels of 100-kb sliding windows that covered entire autosomes. Both raw mCG level-based and ICA-based clustering revealed not only the expected proximity between iRPE and cRPE, but also an apparently counterintuitive

proximity between iPSCs and nRPE (Figure S2A). We suspected that PMDs were potentially responsible for this proximity between iPSCs and nRPE, because both iPSCs and nRPE lacked PMDs (Figures 1B and S1B). Indeed, the proximity was resolved when the genomic regions corresponding to PMDs in fibroblasts (i.e., the PMD-prone regions) were masked in clustering (Figure S2A). Conversely, the proximity was recapitulated in the clustering using the PMD-prone regions (Figure S2A). Thus, we concluded that the counterintuitive proximity between iPSCs and nRPE can be attributed to their lack of PMDs. To minimize the effects of PMDs, we used the methylation levels of distinct genomic features in the following clustering analysis.

We first conducted clustering using the mean mCG levels of promoters or genomic regions within 2 kb upstream of transcription start sites (TSSs). Whereas the ICA-based clustering accurately classified the 16 samples into the expected 6 cell types, the other two approaches regarded one of the nRPE samples (nRPE1) as a cRPE (Figures 2A and S2B). Nevertheless, all types of RPEs (iRPE, xRPE, cRPE, and nRPE) formed a monophyletic clade that excluded the non-RPE cells (iPSCs and fibroblasts), regardless of the clustering method used. The nearest neighbor of iRPE/xRPE was identified as cRPE in ICA-based clustering, whereas it was identified as cRPE/nRPE in the two other approaches (Figures 2A and S2B). Thus, promoter methylation appeared to accurately reflect the status of cellular differentiation. In this context, independent component (IC) 12 was particularly intriguing because it clearly distinguished all types of RPE from the non-RPE cells (Figure 2B). The promoters making large contributions to IC12 were significantly enriched for those of RPE signature genes (Booij et al., 2010; Liao et al., 2010; Strunnikova et al., 2010) (Fisher's exact test, $p = 6.1 \times 10^{-6}$; odds ratio = 7.8) (Figures 2C and S2C).

Next, we performed clustering using the mean mCG levels of CGIs. Here, the 16 samples were accurately classified into the expected six cell types with all three approaches (Figures 2D and S2D). Notably, clustering using CGIs divided the samples into two clusters, one composed of natural or non-reprogrammed cells (fibroblasts, cRPE, and nRPE), and the other comprising cells that had experienced reprogramming (iPSCs, iRPE, and xRPE), regardless of the clustering approach used (Figures 2D and S2D). Here IC14 was noteworthy because it clearly discriminated between the non-reprogrammed and reprogrammed cells (Figure 2E). Major contributors to IC14 were enriched for CGIs proximal to genes involved in homophilic cell adhesion ($p < 1.0 \times 10^{-30}$) (Figure 2F and Table S2).

Finally, we used the mean mCG levels of gene bodies (i.e., the mCG levels of exons and introns) for the clustering analysis. The ICA-based clustering accurately classified the 16 samples into the 6 cell types (Figure S2E), whereas the other two approaches again misclassified nRPE1 as a cRPE sample (Figure S2F). Notably, clustering using gene bodies not only failed to exclude iPSCs from the monophyletic clade including all RPEs, but also identified iPSCs as the nearest neighbor of iRPE/xRPE (Figures S2E and S2F). Since clustering using CGIs also identified iPSC as the nearest neighbor of iRPE/xRPE (Figure 2D), we sought to ascertain whether the CGIs in gene bodies might contribute to the apparent proximity of iPSCs and iRPE/xRPE. Accordingly, we focused on IC5, which discriminated between the reprogrammed and non-reprogrammed cells (Figure S2E). The major contributors to IC5 notably exhibited high CGI density (Figure S2G). Conversely, when CGIs were masked, the proximity between iPSCs and iRPE/xRPE was resolved (Figure S2H). Therefore, CGIs appeared to be responsible for the proximity of iPSCs and iRPE/xRPE in clustering using gene bodies. We were also intrigued by IC4, which largely distinguished between the cells cultivated *in vitro* (fibroblasts, iPSCs, iRPE, and cRPE) and those derived from *in vivo* (xRPE and nRPE) (Figure S2E). However, major contributors to IC4 failed to exhibit enrichment of any specific function (data not shown). In the same context, it is notable that raw mCG level-based clustering using RPE signature gene bodies clearly distinguished between the cells derived from the *in vitro* and *in vivo* settings (Figure S2I). Thus, we assumed that gene-body methylation was partly sensitive to the cellular environment.

Collectively, the clustering results indicated that methylation patterns of individual genomic features reflect distinct aspects of cellular states, including differentiation, experience of reprogramming, and environment. The iRPE methylome supported proper differentiation from iPSCs to RPE, but concurrently exhibited deviation from the nRPE methylome, including the remnant of iPSC-like methylation patterns.

Origin and Fate of Genomic Regions Differentially Methylated between iRPE and nRPE

Following the analysis of global methylation patterns, we focused on differentially methylated regions (DMRs) between iRPE and nRPE, and identified the DMRs using the software metilene (Jühling et al., 2016). To eliminate the effects of sex difference, we excluded both chromosomes X and Y from the analysis. Consequently, we identified 3,020 and 1,906 genomic regions that were hypermethylated and hypomethylated in iRPE relative to nRPE, respectively, which we hereafter refer to as hyper-DMRs and hypo-DMRs (Tables S3 and S4). The hyper-DMRs and hypo-DMRs comprised 2.81 and 3.51 Mb, or 0.10% and 0.12%, of the autosomal genome, respectively, which means that ~99.8% of the autosomal methylome was indistinguishable between iRPE and nRPE under the

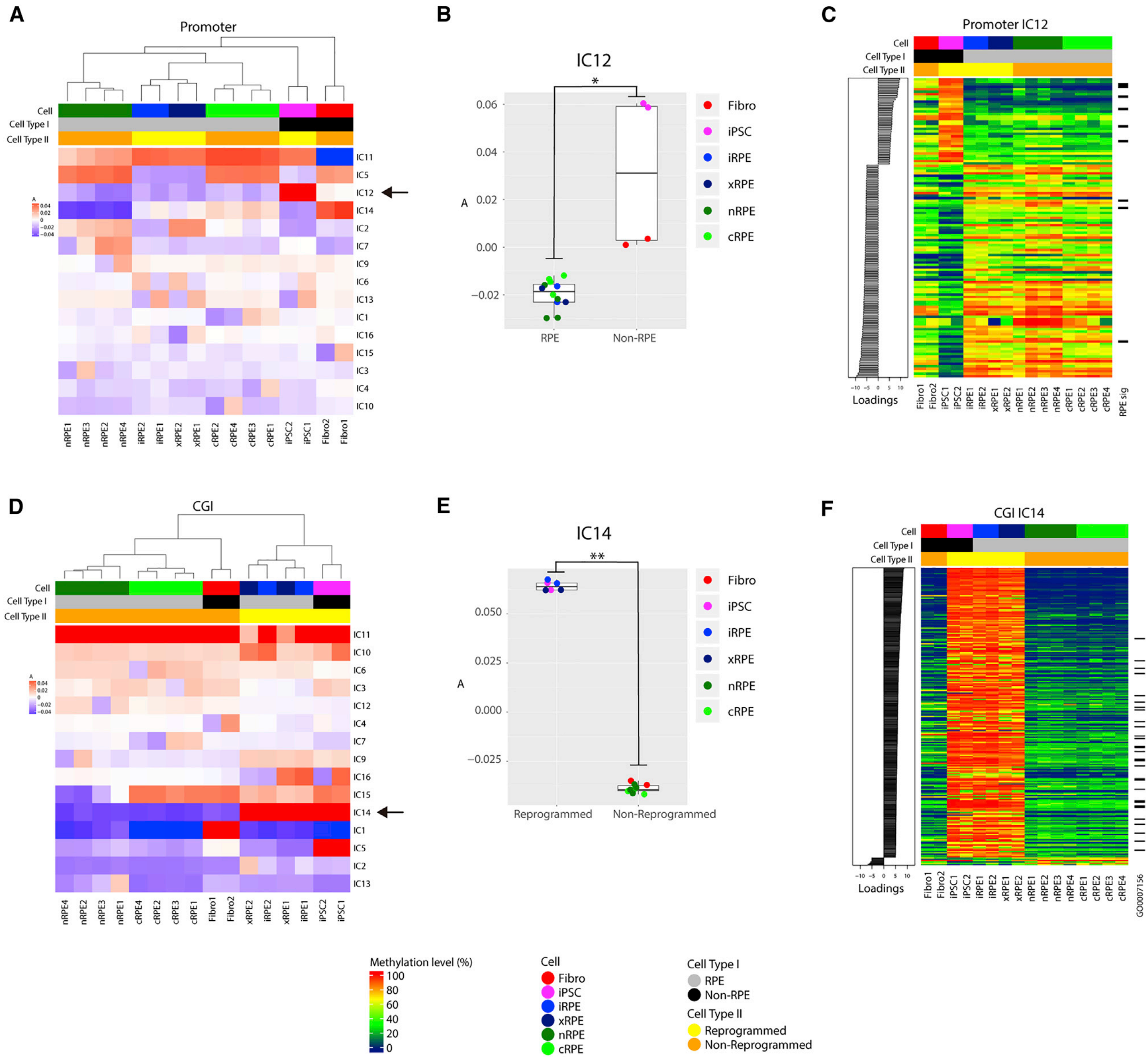

Figure 2. Genomic Feature-Dependent Differential Clustering of iRPE Methylome

(A) ICA-based clustering using the methylation levels of promoters. The color scale indicates element values of the mixing matrix A of ICA (Hyvarinen and Oja, 2000). The columns of A are used for clustering of samples. Note that the nearest neighbor of iRPE/xRPE is cRPE. The arrow indicates the row of A corresponding to IC12.

(B) Differential weighting of IC12 between RPE and non-RPE cells. Element values in the row of A corresponding to IC12 are plotted for individual samples (*p < 0.01 by Wilcoxon test).

(C) Methylation heatmap of promoters with significant contributions to IC12 (absolute value of loadings ≥5). Promoters of RPE signature genes are indicated on the right of the heatmap (RPE sig).

(D) ICA-based clustering using the methylation levels of CGIs. The color scale indicates element values in the mixing matrix A of ICA (Hyvarinen and Oja, 2000). The columns of A are used for clustering of samples. Note that the nearest-neighbor of iRPE/xRPE is iPSC. The arrow indicates the row of A corresponding to IC14.

(E) Differential weighting of IC14 between reprogrammed and non-reprogrammed cells. Element values in the row of A corresponding to IC14 are plotted for individual samples (**p < 0.001 by Wilcoxon test).

(F) Methylation heatmap of CGIs with significant contributions to IC14 (absolute value of loadings ≥5). CGIs proximal to genes involved in "homophilic cell adhesion via plasma membrane adhesion molecules" are indicated on the right of heatmap (GO: 0007156).

criteria used (q value < 0.01; methylation difference >30%; number of CpGs ≥20). These results reinforced the close resemblance between the two methylomes.

We next sought to examine the etiology of the DMRs by tracing their methylation status back in iPSCs. If the cognate region of a DMR was similarly methylated in iPSCs and ESCs, we concluded that the region was properly reprogrammed in iPSCs and that an error in its (de)methylation during differentiation resulted in the DMR. Conversely, if the cognate region of a DMR was differentially methylated between iPSCs and ESCs, and if the methylation status in iPSCs was transmitted to iRPE, we concluded that the DMR could be attributed to aberrant methylation upon reprogramming followed by a failure in its correction during differentiation. Moreover, we traced the fates of DMRs after xenotransplantation to predict their behaviors after autologous transplantation.

We used k-means clustering to classify the 3,020 hyper-DMRs into 5 clusters (clusters 1–5) and then performed hierarchical clustering on each of them to generate a methylation heatmap (Figure 3A). Although most if not all hyper-DMRs were similarly hypermethylated in iPSCs and ESCs, they were demethylated in nRPE but not iRPE. These results indicated that most hyper-DMRs had originated from demethylation errors during differentiation from iPSCs to iRPE rather than errors at reprogramming from fibroblasts to iPSCs. In terms of the fates of the hyper-DMRs, clusters 4 and 5 were particularly interesting because they were demethylated upon xenotransplantation, thus approaching the status of nRPE (Figure 3A). Intriguingly, these regions were largely depleted of CGIs, and this was in sharp contrast with clusters 1–3, whose hypermethylation was retained in xRPE (Figure 3A). Genes proximal to clusters 4 and 5 were notably enriched for RPE signature genes, genes involved in visual perception, and genes downregulated in iRPE relative to nRPE (Figures 3A and 3C). Accordingly, the genes exhibited enrichment for the binding motif of Otx2, a master regulator critically involved in RPE function (Housset et al., 2013) (Figure 3D). By contrast, genes proximal to clusters 1–3 were enriched for those encoding proteins involved in homophilic cell adhesion, such as protocadherin gene clusters (Figure 3C). An RRBS study also reported hypermethylation of these genes in ESC- and iPSC-derived RPE relative to fetal RPE cell lines (Liu et al., 2014).

We similarly examined the 1,906 hypo-DMRs (Figure 3B). Hypo-DMRs were divided into 2 clusters (clusters 6 and 7). The larger cluster (cluster 6) comprised 74% of the hypo-DMRs and showed comparable hypermethylation in iPSCs and ESCs. Because these regions were retained in a hypermethylated state in nRPE but not iRPE, the hypo-DMRs in cluster 6 could likely be attributed to errors in maintenance methylation during differentiation. These hypo-DMRs were generally larger than hyper-DMRs, enriched for LADs to overlap with PMDs, and retained in a hypomethylated state after xenotransplantation (Figure 3B). Conversely, the smaller cluster (cluster 7) comprised 26% of the hypo-DMRs and was hypomethylated in both iPSCs and ESCs. Because these regions acquired intermediate levels of methylation in nRPE but not iRPE, the hypo-DMRs in cluster 7 could be attributed to errors in *de novo* methylation during differentiation. These hypo-DMRs were enriched for CGIs and proximal to genes involved in negative regulation of neural development (Figure 3C). Because these regions were also hypomethylated in cRPE, their hypomethylation in iRPE might have been induced by *in vitro* cultivation and could be mitigated *in vivo*. However, xenotransplantation failed to alter the hypomethylated status of these DMRs (Figure 3B).

Reprogramming-Induced Aberrant Methylation Persistent in iRPE

The results described above indicated that most DMRs could be attributed to (de)methylation errors during differentiation. However, some of the DMRs had clearly originated from aberrant methylation at reprogramming. For instance, eight of the nine reprogramming-associated epigenetic signature genes, which are frequently hypermethylated in iPSCs, but not ESCs (Ruiz et al., 2012), were heavily methylated at their promoter regions in both iPSCs and iRPE in either or both of the two patients (Figure S3A). Moreover, seven out of these eight genes were among the top 1% contributors to IC5 of promoter clustering, which distinguished between the reprogrammed and non-reprogrammed cells (Figures 2A and S3B). The genes were hypermethylated at their CGIs upon reprogramming, and the hypermethylation was maintained throughout differentiation and xenotransplantation, which led to their silencing, as exemplified by *DPP6* (Figures 4A and S3C).

Among the signature genes, *PTPRT* was noteworthy because its promoter CGI was hypermethylated in the reprogrammed cells derived from patient 1 but not patient 2 (Figure 4B). At the *PTPRT* locus, differential methylation occurred not only at the promoter CGI but also in its flanking regions; the regions on both sides of the CGI displayed compromised methylation levels to comprise PMDs in iRPE1, whereas the regions were fully methylated in iRPE2 (Figure 4B). These findings prompted us to examine whether other CGIs also showed a similar pattern, and we focused on IC14 and IC5 of CGI clustering (Figure 2C). CGIs that have major contribution to IC14 were hypermethylated upon reprogramming but failed to be properly demethylated during differentiation. By contrast, major contributors to IC5 were hypermethylated upon reprogramming and successfully demethylated during differentiation. We selected the top 50 CGIs that positively

A

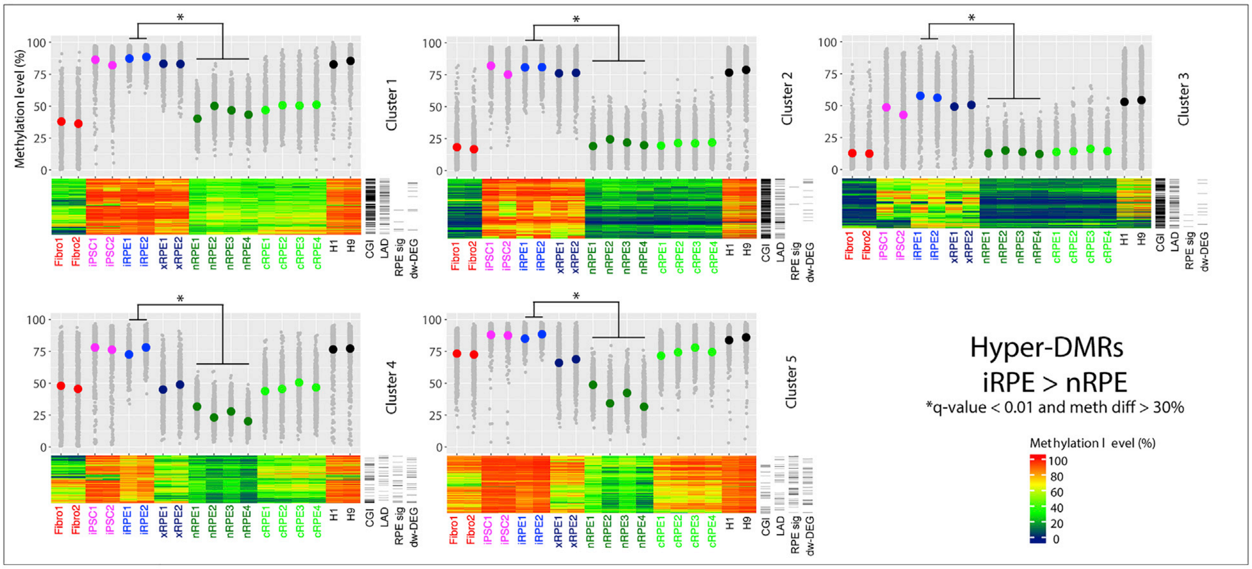

B

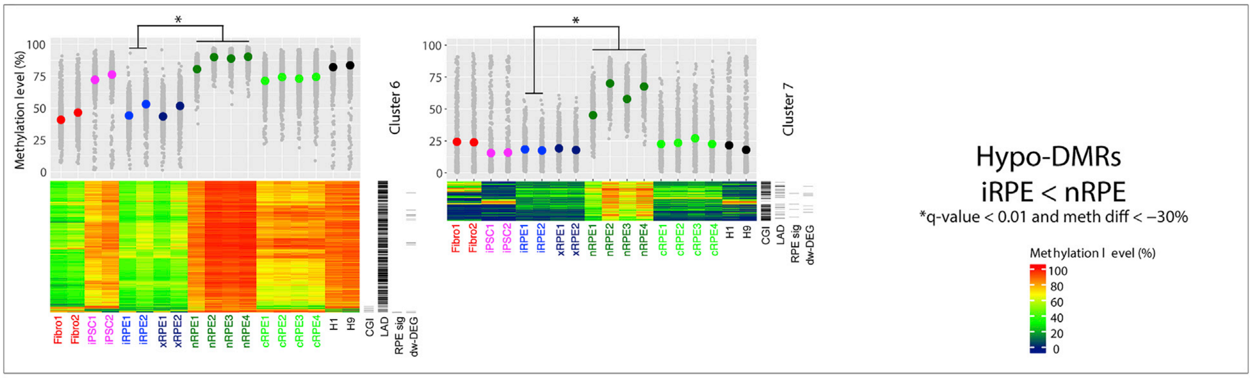

C

DMR	Cluster	GO	P-value
Hyper-DMRs	1	homophilic cell adhesion via plasma membrane adhesion molecules	< 1e-30
		calcium-dependent cell-cell adhesion via plasma membrane cell adhesion molecules	1.3e-06
	2	homophilic cell adhesion via plasma membrane adhesion molecules	< 1e-30
		calcium-dependent cell-cell adhesion via plasma membrane cell adhesion molecules	9.7e-10
		synapse assembly	1.6e-06
	3	homophilic cell adhesion via plasma membrane adhesion molecules	4.3e-16
	4	circadian regulation of gene expression	4.3e-06
	5	visual perception	2.6e-06
		circadian regulation of gene expression	5.0e-06
Hypo-DMRs	7	negative regulation of neuron differentiation	6.6e-06

D

DMR	Cluster	TF	Motif	P-value
Hyper-DMRs	1	NF1	CTTGGCACTGTGCCA	1.0e-08
	4	NF1	CTTGGCACTGTGCCA	1.0e-16
		Tlx	CTGGCAGCCTGCCA	1.0e-09
		GSC	GGGATTAG	1.0e-08
		FOXM1	TGTTTACTTA	1.0e-08
		Otx2	GTTAATCCCT	1.0e-07
	5	NF1	CTTGGCACTGTGCCA	1.0e-09
		Otx2	GTTAATCCCT	1.0e-07
Hypo-DMRs	7	TEA D2	CCTGGAATGT	1.0e-13
		TEA D	CCTGGAATGC	1.0e-12
		TEA D4	CCTGGAATGC	1.0e-11

Figure 3. Tracing the Origin and Fate of DMRs between iRPE and nRPE

(A) Clustering of hyper-DMRs. The 3,020 hyper-DMRs are classified into five clusters (clusters 1–5). Methylation level and heatmap are shown for each cluster in the upper and lower panels, respectively. CGI and LAD indicate DMRs overlapped with CGIs and LADs, respectively. RPE sig and dw-DEG indicate hyper-DMRs within 5 kb from RPE signature genes and genes downregulated in iRPE relative to nRPE, respectively. The panels also include data on human ESC lines H1 and H9 (Lister et al., 2009).

(B) Clustering of hypo-DMRs. The 1,906 hypo-DMRs are classified into two clusters (clusters 6 and 7) and displayed similarly to (A).

(C) GO enrichment analysis of DMR clusters.

(D) Transcription factor-binding motif enrichment analysis of DMR clusters.

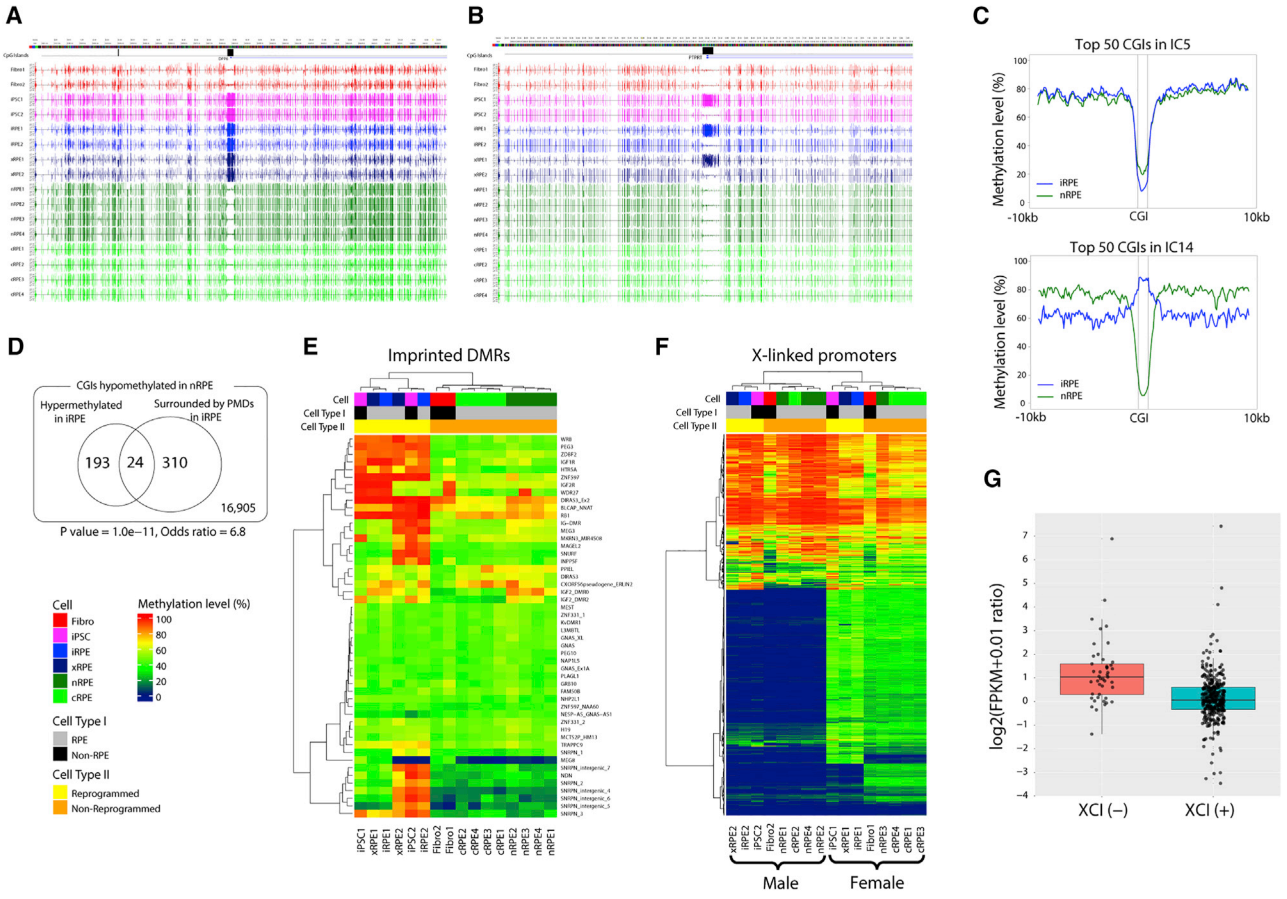

Figure 4. Persistence of Reprogramming-Induced Methylation Errors in iRPE

(A) Genome browser shot of *DPP6*. From top to bottom, tracks represent CGIs, gene models, and mCG levels of both strands in Fibro1, 2; iPSC1, 2; iRPE1, 2; xRPE1, 2; nRPE1–4; and cRPE1–4. Note that promoter CGI is heavily methylated in iPSC, iRPE, and xRPE in both patient 1 and patient 2.

(B) Genome browser shot of *PTPRT*. From top to bottom, tracks represent CGIs, gene models, and mCG levels of both strands in Fibro1, 2; iPSC1, 2; iRPE1, 2; xRPE1, 2; nRPE1–4; and cRPE1–4. Note that promoter CGI is heavily methylated in iPSC, iRPE, and xRPE from patient 1 but not patient 2.

(C) Emergence of PMDs around hypermethylated CGIs. Average methylation profile is shown for the top 50 CGIs contributing to IC5 and IC14 in ICA-based clustering of CGIs (Figure 2C).

(D) Overlap between hypermethylated CGIs and CGIs surrounded by PMDs (Fisher's exact test, $p = 1.8 \times 10^{-11}$).

(E) Methylation heatmap of 50 imprinted DMRs.

(F) Methylation heatmap of X-linked gene promoters. Rows include 1,061 TSS-proximal regions in 773 X-linked genes with enough WGBS coverage, of which 493 (47%) regions in 394 genes appear to subject to XCI.

(G) Expression levels of candidate XCI genes. Log2 value of the expression ratio between iRPE1 and cRPE4 ($[FPKM +0.01]_{iRPE1}/[FPKM +0.01]_{cRPE4}$) is shown for 45 and 349 genes, which are likely escaping from and subject to XCI in iRPE1, being labeled as XCI (−) and XCI (+), respectively.

contributed to IC14 and IC5, and plotted the methylation levels of these CGIs and their flanking regions. The regions flanking IC5-contributing CGIs were comparably hypermethylated between iRPE and nRPE, whereas the regions flanking IC14-contributing CGIs were significantly hypomethylated in iRPE relative to nRPE (Figure 4C). Indeed, co-occurrence of the hypermethylation of a CGI and the hypomethylation of the regions flanking the CGI was statistically significant (Fisher's exact test, $p = 1.0 \times 10^{-11}$; odds ratio = 6.8) (Figure 4D). We thus suspected a mechanistic linkage between the two methylomic alterations, presumably similar to the one recently demonstrated for extraembryonic lineages and cancers (Lorincz and Schübeler, 2017; Smith et al., 2017).

We next examined imprinted genes because reprogramming frequently disrupts genomic imprinting (Liang and

Zhang, 2013; Huo et al., 2014; Bar et al., 2017). We focused on the methylation status of 50 imprinted DMRs (iDMRs) listed in the most comprehensive study conducted on humans thus far (Court et al., 2014). As expected, almost all iDMRs displayed intermediate methylation levels in the non-reprogrammed cells (fibroblasts, cRPE, and nRPE) (Figure 4E). However, 6 iDMRs were hypermethylated in the reprogrammed cells (iPSCs, iRPE, and xRPE) derived from both patients, and an additional 13 iDMRs in 3 imprinted loci showed hypermethylation in the reprogrammed cells derived from patient 2. Thus, hypermethylation occurred at 12% and 38% of the iDMRs in iRPE1 and iRPE2, respectively. For instance, *PEG3* was fully methylated at its promoter CGI and was transcriptionally silenced in iRPE (Figure S4A). Conversely, *ZDBF2* was hypermethylated at its upstream iDMR in iRPE, but its expression was higher in iRPE than in cRPE and nRPE (Figure S4A). A similar gain of iDMR methylation associated with an elevated mRNA level of *ZDBF2* was reported for a case of Temple syndrome (Kagami et al., 2017).

Finally, we examined the status of X chromosome inactivation (XCI) in patient 1 because XCI is frequently eroded in human pluripotent stem cells and their derivatives (Liang and Zhang, 2013; Huo et al., 2014; Pasque and Plath, 2015; Patel et al., 2017). We used the WGBS data of the non-reprogrammed RPE cells (nRPE and cRPE) to identify 394 candidates for genes subject to XCI (XCI genes), in which the TSS-proximal regions (TSS ± 500 bp) displayed low ($\leq$10%) and intermediate (20%–45%) methylation levels in the male and female cells, respectively (Figure S4B). These XCI genes comprised 51% of X-linked genes (394 of the 773 genes with enough WGBS coverage) and significantly overlapped with previously reported XCI genes (Balaton et al., 2015; Tukiainen et al., 2017) (Figure S4C), and most TSS-proximal regions of these genes (87%) overlapped with CGIs (Figure S4B). We next compared the methylation levels of the genes between the reprogrammed and non-reprogrammed female cells. Notably, 45 out of 394 gene promoters (11%) showed prominent hypomethylation ($\leq$5%) in iPSC1, iRPE1, and xRPE1, which indicated their escape from XCI (Figure 4F). For instance, the promoter CGI of *MST4* showed ~50% methylation level in fibroblasts derived from patient 1 and other female control RPE samples, but was almost fully demethylated in the patient's iPSCs, iRPE, and xRPE (Figure S4D). As expected, the 45 genes apparently escaping from XCI were approximately 2-fold derepressed in iRPE1 compared with cRPE4 (Wilcoxon's test, $p = 3.1 \times 10^{-9}$), whereas the remaining 349 genes displayed largely comparable expression levels between the two samples (Figure 4G). These results collectively illustrated a significant erosion of XCI and thus an impairment of dosage compensation in iRPE1.

Correlation between Differential Methylation and Gene Expression

To investigate the overall effects of differential methylation on gene expression, we examined the correlation between DMRs and differentially expressed genes (DEGs) using iRPE1, nRPE1, and nRPE2, because both WGBS and RNA sequencing (RNA-seq) data were available for these samples. We analyzed the WGBS data using metilene (Jühling et al., 2016) under the same criteria as above (Figures 3A and 3B) to reveal 1,783 hyper-DMRs and 638 hypo-DMRs between iRPE1 and nRPE1/2. We then identified 1,254 and 205 genes proximal to, or within 5 kb from, 1,200 hyper-DMRs and 321 hypo-DMRs, respectively. No proximal genes were identified for 583 hyper-DMRs and 317 hypo-DMRs, whereas 32 genes were proximal to both hyper-DMRs and hypo-DMRs. We also analyzed the RNA-seq data using EdgeR (Robinson et al., 2010) to identify DEGs, which revealed 144 and 303 genes upregulated and downregulated in iRPE1 relative to nRPE1/2, respectively; these genes are hereafter referred to as up-DEGs and down-DEGs. Thus, we identified 1,427 (=1,254 + 205 – 32) DMR-proximal genes and 447 (=144 + 303) DEGs in total (Figure 5A; Table S5). These two sets of genes overlapped markedly: overlap was statistically significant between down-DEGs and genes proximal to hyper-DMRs (Fisher's exact test, $p = 3.7 \times 10^{-13}$; odds ratio 3.3) and between up-DEGs and genes proximal to hypo-DMRs (Fisher's exact test, $p = 1.0 \times 10^{-8}$; odds ratio 9.1) (Figure S5A).

We next examined whether genes with specific functions were enriched among DMR-proximal DEGs. Intriguingly, 59 down-DEGs proximal to hyper-DMRs exhibited an enrichment of genes related to visual perception (Figure 5B). One of these genes, *RLBP1*, encodes the cellular retinaldehyde-binding protein, which is critically involved in the visual cycle; this metabolic pathway regenerates 11-*cis*-retinal from all-*trans*-retinal generated upon photon absorption (Kiser et al., 2012) and was demonstrated to be functional in iRPE both *in vitro* and *in vivo* (Maeda et al., 2013). The finding on *RLBP1* led us to inspect the methylation status of other visual-cycle genes, and we revealed hypermethylation at their gene bodies in iRPE compared with nRPE, as exemplified by *RPE65* (Figure S5B). Among the five visual-cycle genes that function in RPE, four genes (*RBP1*, *RPE65*, *RLBP1*, and *RDH5*) exhibited inverse correlations between the levels of gene-body methylation and mRNA abundance in iRPE1, cRPE2/4, and nRPE1/2 (Figure 5C). Notably, these genes showed demethylation upon xenotransplantation (Figure S5C). From the inverse correlations and methylation levels in xRPE, we might expect 3.5- to 7.8-fold induction of these genes after xenotransplantation, although unavailability of relevant RNA samples precluded direct examination of this

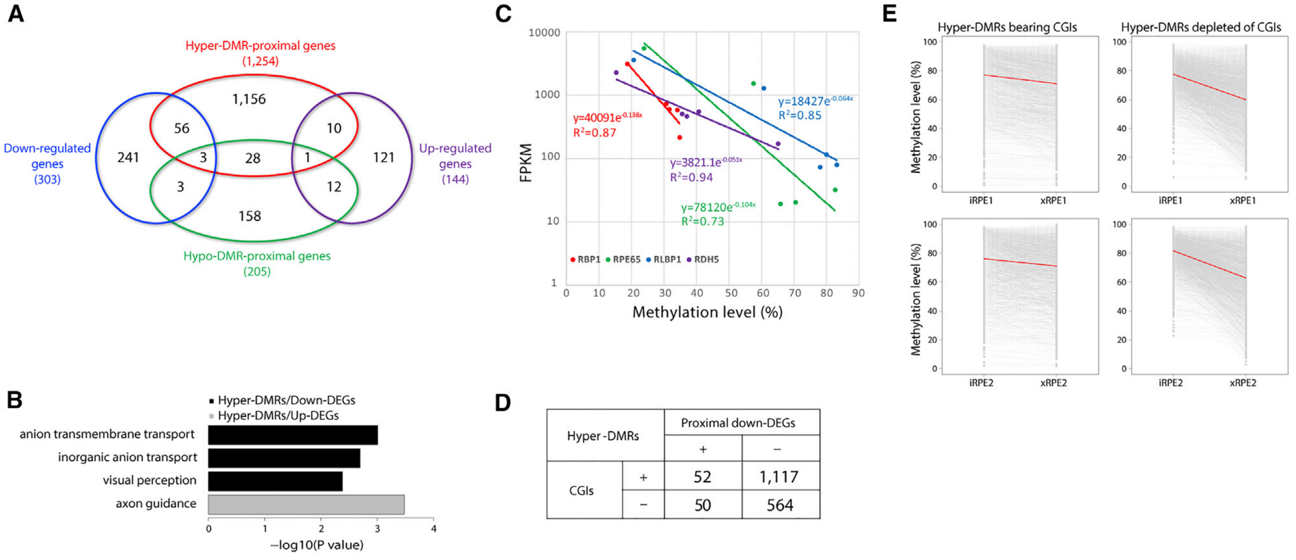

Figure 5. Correlation between DMRs and DEGs

(A) Venn diagram for DMR-proximal genes and DEGs. Note that DMRs and DEGs used in this analysis are identified between iRPE1 and nRPE1/2.

(B) GO enrichment analysis of DEGs proximal to hyper-DMRs.

(C) Correlation between gene-body methylation and expression in four visual-cycle genes.

(D) Co-occurrence of CGIs and proximal down-DEGs in hyper-DMRs (Fisher's exact test, $p = 1.8 \times 10^{-3}$).

(E) Ladder charts for methylation levels of hyper-DMRs in iRPE and xRPE. Hyper-DMRs are divided into those bearing and depleted of CGIs. Red lines indicate the mean methylation change.

prediction (Figure S5C). The remaining gene, *LRAT*, harbored a hypo-DMR in the upstream flanking region of its promoter CGI, whose methylation level was inversely correlated with the expression level of *LRAT* mRNA (Figure S5D).

The observations on the visual-cycle genes prompted us to examine the origin and fate of DEG-proximal DMRs. The methylation heatmaps suggested that hyper-DMRs proximal to down-DEGs were concentrated to clusters 4 and 5, which were largely depleted of CGIs, amenable to xenotransplantation-induced demethylation, and enriched for proximal genes involved in visual perception (Figure 3A). Accordingly, we detected significant overlap between hyper-DMRs proximal to down-DEGs and those depleted of CGIs (Fisher's exact test, $p = 1.8 \times 10^{-3}$) (Figure 5D). Hyper-DMRs depleted of CGIs appeared to be more prone to xenotransplantation-induced demethylation than hyper-DMRs bearing CGIs (Figure 5E). These observations on hyper-DMRs suggested a correlation among the presence of proximal down-DEGs, lack of CGIs, and xenotransplantation-induced demethylation. *In vivo* conditions after xenotransplantation might have either mitigated hypermethylation of hyper-DMRs to derepress proximal down-DEGs or derepressed down-DEGs to induce demethylation of proximal hyper-DMRs.

Transplantation-Induced Restoration of Non-CpG Methylation Missing in iRPE

As well as mCG, we also examined the status of non-CpG methylation (mCH; H = A, C, or T) (Figures 6A and 6B). As expected from previous studies (Lister et al., 2009, 2011), DNA methylation occurred almost exclusively at CG sites in fibroblasts, whereas iPSCs displayed notable levels of mCHs, particularly in the context of CHG (Figures 6A and 6B). Conversely, nRPE unexpectedly harbored a high level of mCHs in the context of both CHG and CHH (Figures 6A and 6B). The mCH level of nRPE1 was lower than those of the other nRPE samples for unknown reasons but still higher than that of fibroblasts (Figure S6A). Previously, CAG and CAC were identified as mCH hotspots in pluripotent stem cells and neural cells, respectively (Lister et al., 2011, 2013; He and Ecker, 2015; Schultz et al., 2015). In our samples, CAG and CAC showed the highest methylation levels in iPSCs and nRPE, respectively (Figure 6C). In nRPE, the other CAs (i.e., CAA, CAG, and CAT) and CTC displayed the second highest mCH levels after CAC, similarly to those in neural cells (Lister et al., 2013) (Figures 6C and S6A). Note that mCHs in ESCs and neurons were shown to correlate positively and negatively with gene expression, respectively (Guo et al., 2014; Lister et al., 2009, 2013). In nRPE, both promoter and gene-body mCH levels were inversely correlated with gene-expression

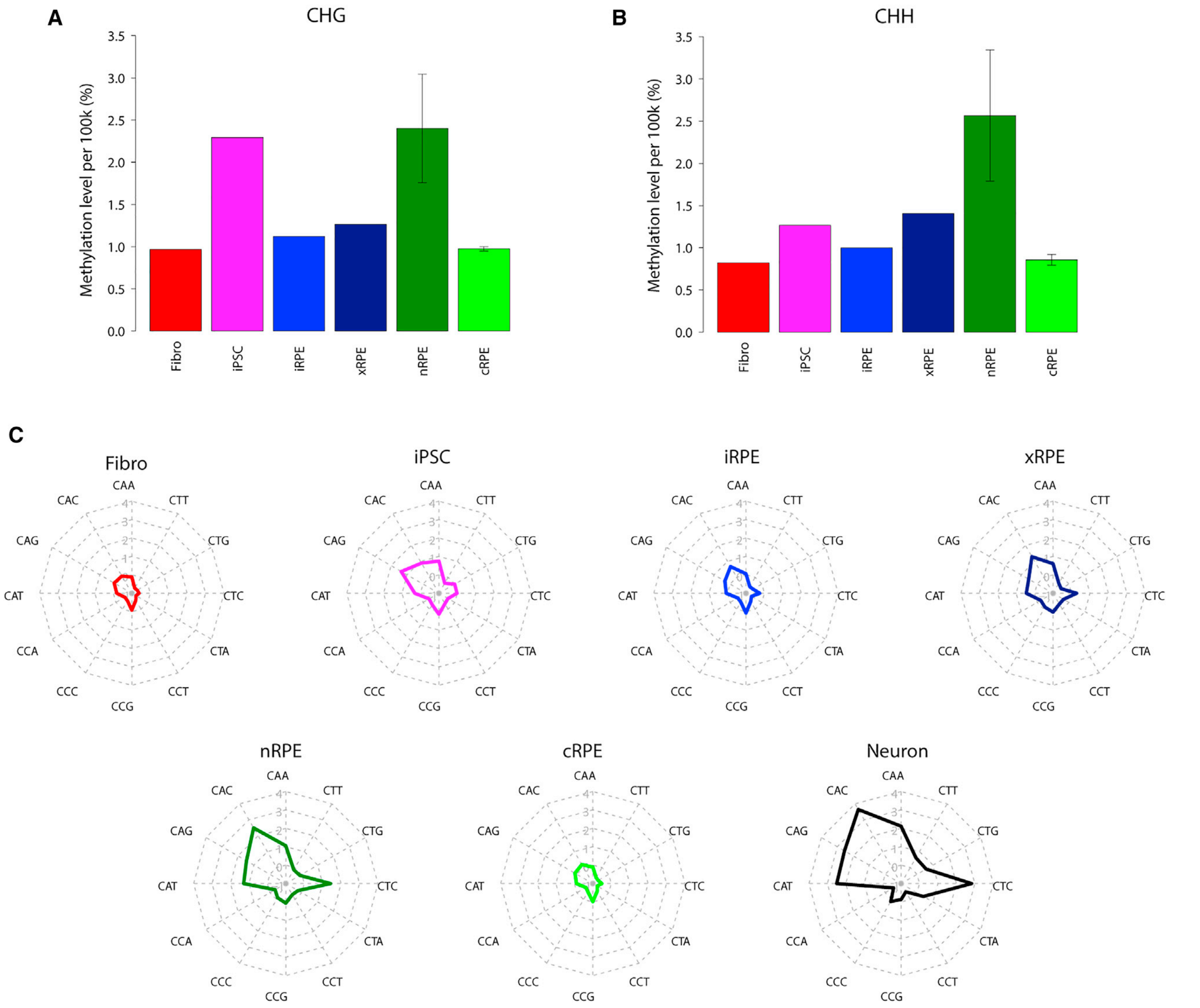

Figure 6. Dynamic Change of Non-CpG Methylation

(A) Mean mCHG levels of each cell type (H = A, C, or T).

(B) Mean mCHH levels of each cell type.

(C) Radar chart for sequence contexts of mCH. Methylation levels are plotted for the 12 possible CHN sequence contexts. The numbers in the left of the CCG axis indicated log2 values of methylation levels (i.e., 1%, 2%, 4%, 8%, and 16%).

levels (Figure S6B). Among the genes featuring high promoter mCH levels in nRPE, we detected an enrichment of genes involved in development and differentiation of mesodermal tissues (Figure S6C).

Notably, iRPE failed to recapitulate the high mCH level, a unique feature of nRPE (Figures 6A, 6B, and S6A). We also noted that cRPE lost mCH (Figures 6A, 6B, and S6A). We thus intended to determine whether *in vitro* cultivation might have caused the lack of mCH in iRPE and whether *in vivo* conditions might increase mCHs. In accordance with this scenario, xRPE exhibited higher mCH levels than iRPE (Figures 6A, 6B, and S6A). Importantly, mCHs in nRPE and xRPE shared an almost identical preference in terms of their sequence contexts (Figure 6C). Moreover, the genomic distributions of mCHs appeared largely comparable between nRPE and xRPE, although the mCH levels of xRPE were not as high as those of nRPE (Figure S6D). Thus, *in vivo* conditions can likely remodel the iRPE methylome and thereby cause mCH accumulation in a physiologically relevant manner.

Taken together, these results suggest that mCHs in RPE share characteristics similar to those of mCHs in neural

cells and can respond to the cellular environment. Importantly, the responsiveness was faithfully recapitulated in iRPE. Therefore, we expect the iRPE sheet in the patient's eye to accumulate mCHs, making its methylome even closer to that of nRPE.

DISCUSSION

The results of base-resolution methylome analyses performed here supported proper differentiation of the iRPE used in the first-in-human trial of iPSC-based autologous transplantation. However, the results also delineated the differences between iRPE and nRPE; emergence of PMDs in iRPE, diverse DMRs between iRPE and nRPE, and the lack of mCHs in iRPE. Because some of these differences were observed even between cRPE and nRPE, they could be attributed to the effects of *in vitro* cultivation and might be mitigated under *in vivo* conditions after transplantation. Indeed, xenotransplantation to mouse remodeled the iRPE methylome to demethylate hyper-DMRs proximal to genes suppressed in iRPE and to accumulate mCHs in a physiologically relevant manner. These results demonstrated the plasticity exhibited by the iRPE methylome in responding appropriately to the *in vivo* environment for further functional maturation. By contrast, PMDs and hypermethylated CGIs were refractory to *in vivo* environment-induced mitigation, at least within the period of observation in this study. It is conceivable that these methylomic alterations are within a clinically acceptable range of epigenetic variations, considering the satisfactory outcome of patient 1. Further investigation is nevertheless required to elucidate the biological and clinical significance of the alterations. It would be also critical to delineate the normal/physiological range of methylomic variations based on WGBS of a much larger number of nRPE for more precise evaluation of iRPE. The iPSC-derived grafts are expected to function properly for an extended period of time in the recipients, during which they would be inevitably exposed to various pathophysiological changes. It is thus ideal that they are comparable with their natural counterparts in their capability of responding to such changes. Since the methylome serves as a good indicator of cellular potential, its sequencing can play an important role in evaluating iPSC-derived grafts. The findings reported here should provide a useful reference for examining the methylomes of various iPSC-derived cells.

EXPERIMENTAL PROCEDURES

Samples

The DNA and RNA samples analyzed in this study were prepared as described previously (Mandai et al., 2017). See Supplemental Information for details.

WGBS

All WGBS libraries were prepared using the PBAT method from ~100 ng of genomic DNA without using any global PCR amplification (Miura et al., 2012; Miura and Ito, 2018). Illumina sequencing was conducted on HiSeq2500 using 100-nt single-read mode. WGBS reads were mapped to the human reference genome sequence (hg19) using Bmap (http://itolab.med.kyushu-u.ac.jp/BMap/). All these experiments and the following analyses were carried out in accordance with our institutional guidelines and were approved by the Ethics Committee for Human Genome and Gene Analysis Research at the Kyushu University Hospital Campus (approval number 626-00). The publicly available WGBS data on human ESC H1 and H9 (Lister et al., 2009) were downloaded from the database and processed similarly for comparison.

RNA-Seq

See Supplemental Information for details.

Bioinformatics Analysis

Methylation Levels

Methylation levels were determined for individual CG and CH sites covered at least once and 10 times, respectively. Methylation levels of sliding windows, promoters, gene bodies, CGIs, and TSS-proximal regions were determined by averaging the methylation levels of CG/CH sites in individual features.

Methylome Segmentation and PMD Detection

Methylome segmentation was conducted using SimpleChangepointCalculator (Yokoyama et al., 2015; http://itolab.med.kyushu-u.ac.jp/CPT/). Global segmentation patterns were visualized as MDL plots, which displays the size, the methylation level, and the number of domains (Yokoyama et al., 2015). Segmented regions that are larger than 10 kb in size and lower than 70% in mean methylation level were defined as PMDs on the basis of the original definition (Lister et al., 2009).

Cluster Analysis

We applied three unsupervised classification methods, namely hierarchical clustering, ICA, and PCA, to mean methylation levels of 100-kb sliding windows, promoters (<2 kb upstream of TSS), gene bodies, and CGIs. Besides the raw mCG levels, we also used the outputs of ICA and PCA to generate the Euclidian distance matrices for hierarchical clustering by Ward's method. In the hierarchical clustering using the outputs of ICA and PCA, the common component was removed. ICA was done using the fastICA package in R, which implements the algorithm of Hyvarinen and Oja (2000). All heatmaps or clustering were performed using the ComplexHeatmaps library in bioconductor (Gu et al., 2016).

Identification of DMRs

DMRs between iRPE and nRPE were identified using metilene with default parameters (Jühling et al., 2016). DMRs were filtered to retain those containing at least 20 CpGs with a q value less than 0.01 and methylation difference larger than 30%. To classify DMRs between iRPE and nRPE based on methylation status in the other samples, we conducted k-means clustering using the function k-means of the amap package in R. We determined the cluster numbers so that characteristic clusters were most evidently highlighted.

Motif Analysis

Motif enrichment analysis for known transcription factor-binding motifs within the DMRs was performed using the findMotifsGenome.pl of HOMER (Heinz et al., 2010) with the "-size given," "-mask," and "-cpg" parameters. The threshold for motif identification was a p value of 1.0×10^{-7}.

Functional Enrichment Analysis

All functional enrichment analysis with gene ontology (GO) biological process terms was performed using the topGO package in R (Alexa and Rahnenfuhrer, 2016). To avoid overly general or specific terms, GO terms annotating more than 500 or fewer than 10 genes were removed. A p value of 0.01 was set to be the significance level for gene sets, with an additional requirement of at least 3 genes with annotated biological functions.

ACCESSION NUMBERS

The accession numbers for the WGBS and RNA-seq data reported in this paper are GEO: GSE120140 and GSE118167, respectively. Because data from the patients include information that can identify the individual, we will consider providing data upon request after appropriate ethical review. We will provide SNP-masked methylation level data upon request.

SUPPLEMENTAL INFORMATION

Supplemental Information can be found online at https://doi.org/10.1016/j.stemcr.2019.08.014.

AUTHOR CONTRIBUTIONS

S.Y., M. Takahashi, and T.I. conceived the project; H.A., F.M., A.W., C.M., F.K., Y.K., N.S., Y.S., M. Terada, and S.G. performed the experiments and analyzed the data; H.A. and T.I. wrote the paper.

ACKNOWLEDGMENTS

We appreciate the technical assistance from The Research Support Center, Research Center for Human Disease Modeling, Kyushu University Graduate School of Medical Sciences. This work was partly supported by the Japan Agency for Medical Research and Development (AMED) under grant numbers JP16am0101059 and JP18am0101103, and by the Japan Society for the Promotion of Science (JSPS) KAKENHI 17H06305.

Received: November 10, 2018
Revised: August 26, 2019
Accepted: August 28, 2019
Published: September 26, 2019

REFERENCES

Alexa, A., and Rahnenfuhrer, J. (2016). topGO: Enrichment Analysis for Gene Ontology. R Package Ver. 2.26.0. https://rdrr.io/bioc/topGO/.

Balaton, B.P., Cotton, A.M., and Brown, C.J. (2015). Derivation of consensus inactivation status for X-linked genes from genome-wide studies. Biol. Sex Differ. *6*, 35.

Bar, S., Schachter, M., Eldar-Geva, T., and Benvenisty, N. (2017). Large-scale analysis of loss of imprinting in human pluripotent stem cells. Cell Rep. *19*, 957–968.

Booij, J.C., ten Brink, J.B., Swagemakers, S.M., Verkerk, A.J., Essing, A.H., van der Spek, P.J., and Bergen, A.A. (2010). A new strategy to identify and annotate human RPE-specific gene expression. PLoS One *5*, e9341.

Court, F., Tayama, C., Romanelli, V., Martin-Trujillo, A., Iglesias-Platas, I., Okamura, K., Sugahara, N., Simón, C., Moore, H., Harness, J.V., et al. (2014). Genome-wide parent-of-origin DNA methylation analysis reveals the intricacies of human imprinting and suggests a germline methylation-independent mechanism of establishment. Genome Res. *24*, 554–569.

Gu, Z., Eils, R., and Schlesner, M. (2016). Complex heatmaps reveal patterns and correlations in multidimensional genomic data. Bioinformatics *32*, 2847–2849.

Guo, J.U., Su, Y., Shin, J.H., Shin, J., Li, H., Xie, B., Zhong, C., Hu, S., Le, T., Fan, G., et al. (2014). Distribution, recognition and regulation of non-CpG methylation in the adult mammalian brain. Nat. Neurosci. *17*, 215–222.

He, Y., and Ecker, J.R. (2015). Non-CG methylation in the human genome. Annu. Rev. Genomics Hum. Genet. *16*, 55–77.

Heinz, S., Benner, C., Spann, N., Bertolino, E., Lin, Y.C., Laslo, P., Cheng, J.X., Murre, C., Singh, H., and Glass, C.K. (2010). Simple combinations of lineage-determining transcription factors prime cis-regulatory elements required for macrophage and B cell identities. Mol. Cell *38*, 576–589.

Housset, M., Samuel, A., Ettaiche, M., Bemelmans, A., Béby, F., Billon, N., and Lamonerie, T. (2013). Loss of Otx2 in the adult retina disrupts retinal pigment epithelium function, causing photoreceptor degeneration. J. Neurosci. *33*, 9890–9904.

Huo, J.S., Baylin, S.B., and Zambidis, E.T. (2014). Cancer-like epigenetic derangements of human pluripotent stem cells and their impact on applications in regeneration and repair. Curr. Opin. Genet. Dev. *28*, 43–49.

Hyvarinen, A., and Oja, E. (2000). Independent component analysis: algorithms and applications. Neural Networks *13*, 411–430.

Inoue, H., Nagata, N., Kurokawa, H., and Yamanaka, S. (2014). iPS cells: a game changer for future medicine. EMBO J. *33*, 409–417.

Jühling, F., Kretzmer, H., Bernhart, S.H., Otto, C., Stadler, P.F., and Hoffmann, S. (2016). metilene: fast and sensitive calling of differentially methylated regions from bisulfite sequencing data. Genome Res. *26*, 256–262.

Kagami, M., Matsubara, K., Nakabayashi, K., Nakamura, A., Sano, S., Okamura, K., Hata, K., Fukami, M., and Ogata, T. (2017). Genome-wide multilocus imprinting disturbance analysis in Temple syndrome and Kagami-Ogata syndrome. Genet. Med. *19*, 476–482.

Kamao, H., Mandai, M., Okamoto, S., Sakai, N., Suga, A., Sugita, S., Kiryu, J., and Takahashi, M. (2014). Characterization of human induced pluripotent stem cell-derived retinal pigment epithelium cell sheets aiming for clinical application. Stem Cell Reports *2*, 205–218.

Kiser, P.D., Golczak, M., Maeda, A., and Palczewski, K. (2012). Key enzymes of the retinoid (visual) cycle in vertebrate retina. Biochim. Biophys. Acta *1821*, 137–151.

Liang, G., and Zhang, Y. (2013). Genetic and epigenetic variations in iPSCs: potential causes and implications for application. Cell Stem Cell *13*, 149–159.

Liao, J.L., Yu, J., Huang, K., Hu, J., Diemer, T., Ma, Z., Dvash, T., Yang, X.J., Travis, G.H., Williams, D.S., et al. (2010). Molecular signature of primary retinal pigment epithelium and stem-cell-derived RPE cells. Hum. Mol. Genet. *19*, 4229–4238.

Lister, R., Pelizzola, M., Dowen, R.H., Hawkins, R.D., Hon, G., Tonti-Filippini, J., Nery, J.R., Lee, L., Ye, Z., Ngo, Q.M., et al. (2009). Human DNA methylomes at base resolution show widespread epigenomic differences. Nature *462*, 315–322.

Lister, R., Pelizzola, M., Kida, Y.S., Hawkins, R.D., Nery, J.R., Hon, G., Antosiewicz-Bourget, J., O'Malley, R., Castanon, R., Klugman, S., et al. (2011). Hotspots of aberrant epigenomic reprogramming in human induced pluripotent stem cells. Nature *471*, 68–73.

Lister, R., Mukamel, E.A., Nery, J.R., Urich, M., Puddifoot, C.A., Johnson, N.D., Lucero, J., Huang, Y., Dwork, A.J., Schultz, M.D., et al. (2013). Global epigenomic reconfiguration during mammalian brain development. Science *341*, 1237905.

Liu, Z., Jiang, R., Yuan, S., Wang, N., Feng, Y., Hu, G., Zhu, X., Huang, K., Ma, J., Xu, G., et al. (2014). Integrated analysis of DNA methylation and RNA transcriptome during in vitro differentiation of human pluripotent stem cells into retinal pigment epithelial cells. PLoS One *9*, e91416.

Lorincz, M.C., and Schübeler, D. (2017). Evidence for converging DNA methylation pathways in placenta and cancer. Dev. Cell *43*, 257–258.

Maeda, T., Lee, M.J., Palczewska, G., Marsili, S., Tesar, P.J., Palczewski, K., Takahashi, M., and Maeda, A. (2013). Retinal pigmented epithelial cells obtained from human induced pluripotent stem cells possess functional visual cycle enzymes in vitro and in vivo. J. Biol. Chem. *288*, 34484–34493.

Mandai, M., Watanabe, A., Kurimoto, Y., Hirami, Y., Morinaga, C., Daimon, T., Fujihara, M., Akimaru, H., Sakai, N., Shibata, Y., et al. (2017). Autologous induced stem-cell-derived retinal cells for macular degeneration. N. Engl. J. Med. *376*, 1038–1046.

Miura, F., Enomoto, Y., Dairiki, R., and Ito, T. (2012). Amplification-free whole-genome bisulfite sequencing by post-bisulfite adaptor tagging. Nucleic Acids Res. *40*, e136.

Miura, F., and Ito, T. (2018). Post-bisulfite adaptor tagging for PCR-free whole-genome bisulfite sequencing. Methods Mol. Biol. *1708*, 123–136.

Pasque, V., and Plath, K. (2015). X Chromosome reactivation in reprogramming and in development. Curr. Opin. Cell Biol. *37*, 75–83.

Patel, S., Bonora, G., Sahakyan, A., Kim, R., Chronis, C., Langerman, J., Fitz-Gibbon, S., Rubbi, L., Skelton, R.J., Ardehali, R., et al. (2017). Human embryonic stem cells do not change their X inactivation status during differentiation. Cell Rep. *18*, 54–67.

Robinson, M.D., McCarthy, D.J., and Smyth, G.K. (2010). edgeR: a Bioconductor package for differential expression analysis of digital gene expression data. Bioinformatics *26*, 139–140.

Ruiz, S., Diep, D., Gore, A., Panopoulos, A.D., Montserrat, N., Plongthongkum, N., Kumar, S., Fung, H.L., Giorgetti, A., Bilic, J., et al. (2012). Identification of a specific reprogramming-associated epigenetic signature in human induced pluripotent stem cells. Proc. Natl. Acad. Sci. U S A *109*, 16196–16201.

Schultz, M.D., He, Y., Whitaker, J.W., Hariharan, M., Mukamel, E.A., Leung, D., Rajagopal, N., Nery, J.R., Urich, M.A., Chen, H., et al. (2015). Human body epigenome maps reveal noncanonical DNA methylation variation. Nature *523*, 212–216.

Smith, Z.D., Shi, J., Gu, H., Donaghey, J., Clement, K., Cacchiarelli, D., Gnirke, A., Michor, F., and Meissner, A. (2017). Epigenetic restriction of extraembryonic lineages mirrors the somatic transition to cancer. Nature *549*, 543–547.

Strunnikova, N.V., Maminishkis, A., Barb, J.J., Wang, F., Zhi, C., Sergeev, Y., Chen, W., Edwards, A.O., Stambolian, D., Abecasis, G., et al. (2010). Transcriptome analysis and molecular signature of human retinal pigment epithelium. Hum. Mol. Genet. *19*, 2468–2486.

Tapia, N., and Schöler, H.R. (2016). Molecular obstacles to clinical translation of iPSCs. Cell Stem Cell *19*, 298–309.

Trounson, A., and DeWitt, N.D. (2016). Pluripotent stem cells progressing to the clinic. Nat. Rev. Mol. Cell. Biol. *17*, 194–200.

Tukiainen, T., Villani, A.C., Yen, A., Rivas, M.A., Marshall, J.L., Satija, R., Aguirre, M., Gauthier, L., Fleharty, M., Kirby, A., et al. (2017). Landscape of X chromosome inactivation across human tissues. Nature *550*, 244–248.

Yokoyama, T., Miura, F., Araki, H., Okamura, K., and Ito, T. (2015). Changepoint detection in base-resolution methylome data reveals a robust signature of methylated domain landscape. BMC Genomics *16*, 594.

Stem Cell Reports, Volume *13*

Supplemental Information

Base-Resolution Methylome of Retinal Pigment Epithelial Cells Used in the First Trial of Human Induced Pluripotent Stem Cell-Based Autologous Transplantation

Hiromitsu Araki, Fumihito Miura, Akira Watanabe, Chikako Morinaga, Fumiyo Kitaoka, Yuko Kitano, Noriko Sakai, Yumiko Shibata, Motoki Terada, So Goto, Shinya Yamanaka, Masayo Takahashi, and Takashi Ito

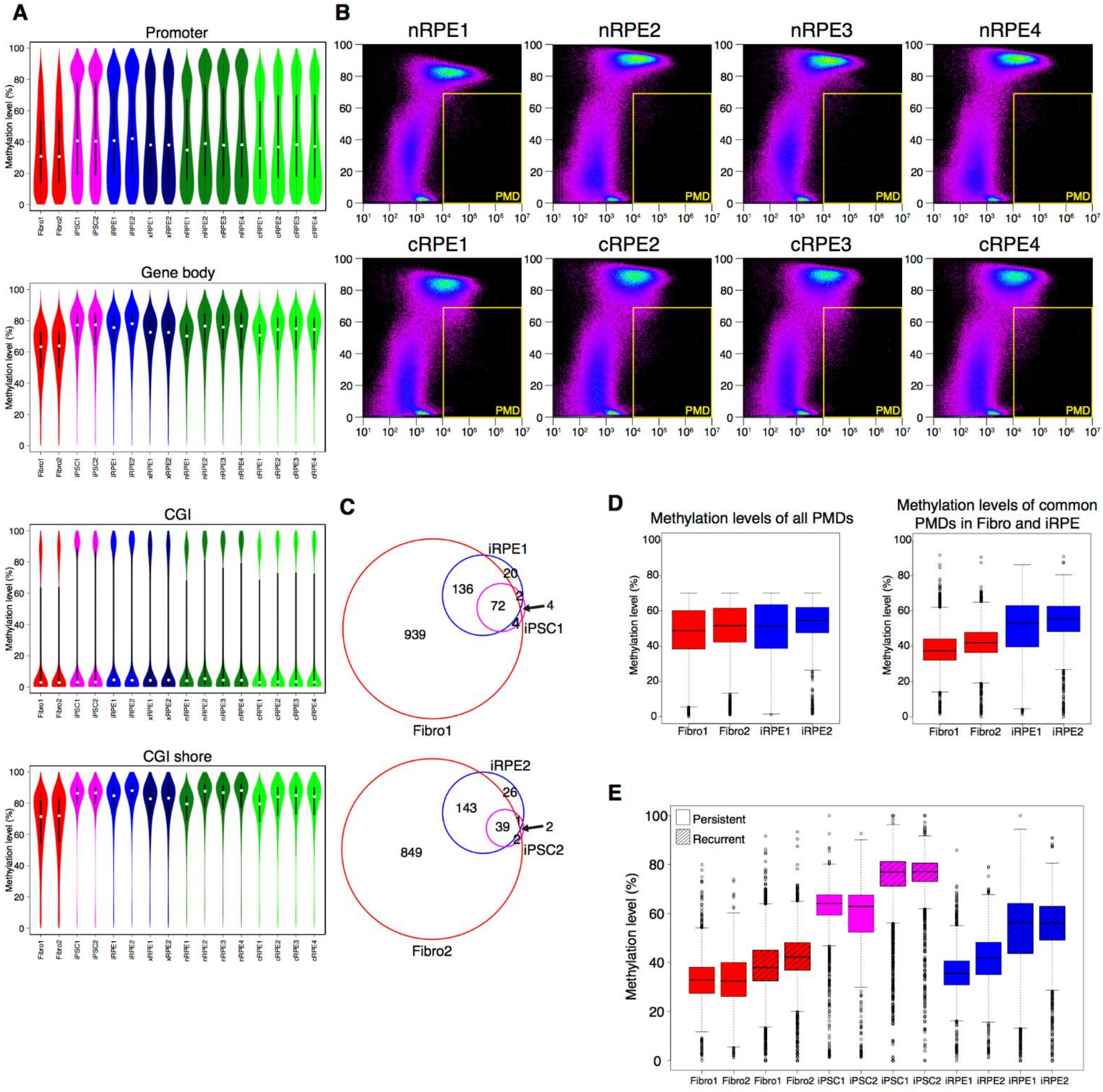

Figure S1. Characterization of PMDs. Related to Figure 1.

(A) Violin plots of mCG levels for indicated genomic features. Promoter, genomic region spanning 2 kb upstream of TSS; CGI shore, genomic region spanning 2 kb upstream and downstream of each CGI.

(B) MDL plots for all nRPE and cRPE samples.

(C) Venn diagram for overlap among PMDs in fibroblasts, iPSCs, and iRPE. The number indicates the total size (Mb) of genomic regions included in each section.

(D) Box plots for mCG levels of all and common PMDs in fibroblasts and iRPE.

(E) Box plots for mCG levels of persistent and recurrent PMDs in fibroblasts, iPSCs, and iRPE.

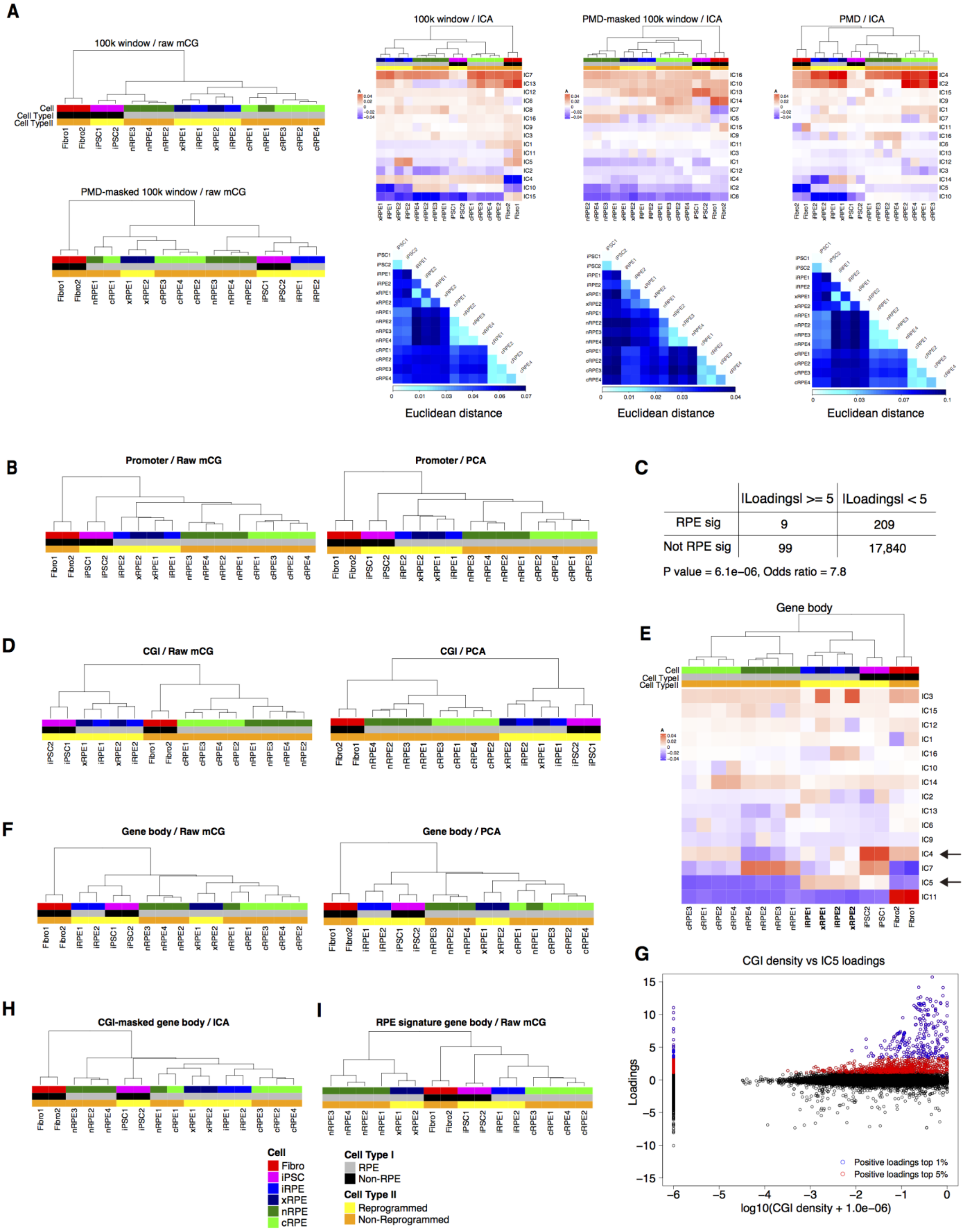

	lLoadingsl >= 5	lLoadingsl < 5
RPE sig	9	209
Not RPE sig	99	17,840

Figure S2. Genomic Feature-Dependent Differential Clustering. Related to Figure 2.

(A) Clustering using 100-kb sliding windows. Results of raw mCG level-based clustering are shown for the whole and PMD-masked genomes (left). Results of ICA-based clustering are shown for the whole genome, PMD-masked genome, and PMDs with color-coded Euclidean distance matrices calculated from the columns of the mixing matrix A of ICA (right). Genomic regions judged as PMDs in fibroblasts are used for masking.

(B) Raw mCG level-based and PCA-based clustering using promoters.

(C) Enrichment of RPE-signature genes in promoters with large contributions to IC12 (absolute value of loadings ≥ 5).

(D) Raw mCG level-based and PCA-based clustering using CGIs.

(E) ICA-based clustering using gene bodies. Arrows indicate the rows corresponding to IC4 and IC5, which show differential weighting between cells in vitro and those in vivo and between reprogrammed and non-reprogrammed cells, respectively.

(F) Raw mCG level-based and PCA-based clustering using gene bodies.

(G) CGI density and factor loadings for IC5 of clustering using gene bodies. CGI density represents the ratio of total nucleotides in CGIs in a gene body to those of the gene body.

(H) ICA-based clustering using CGI-masked gene bodies.

(I) Raw mCG level-based clustering using RPE-signature gene bodies.

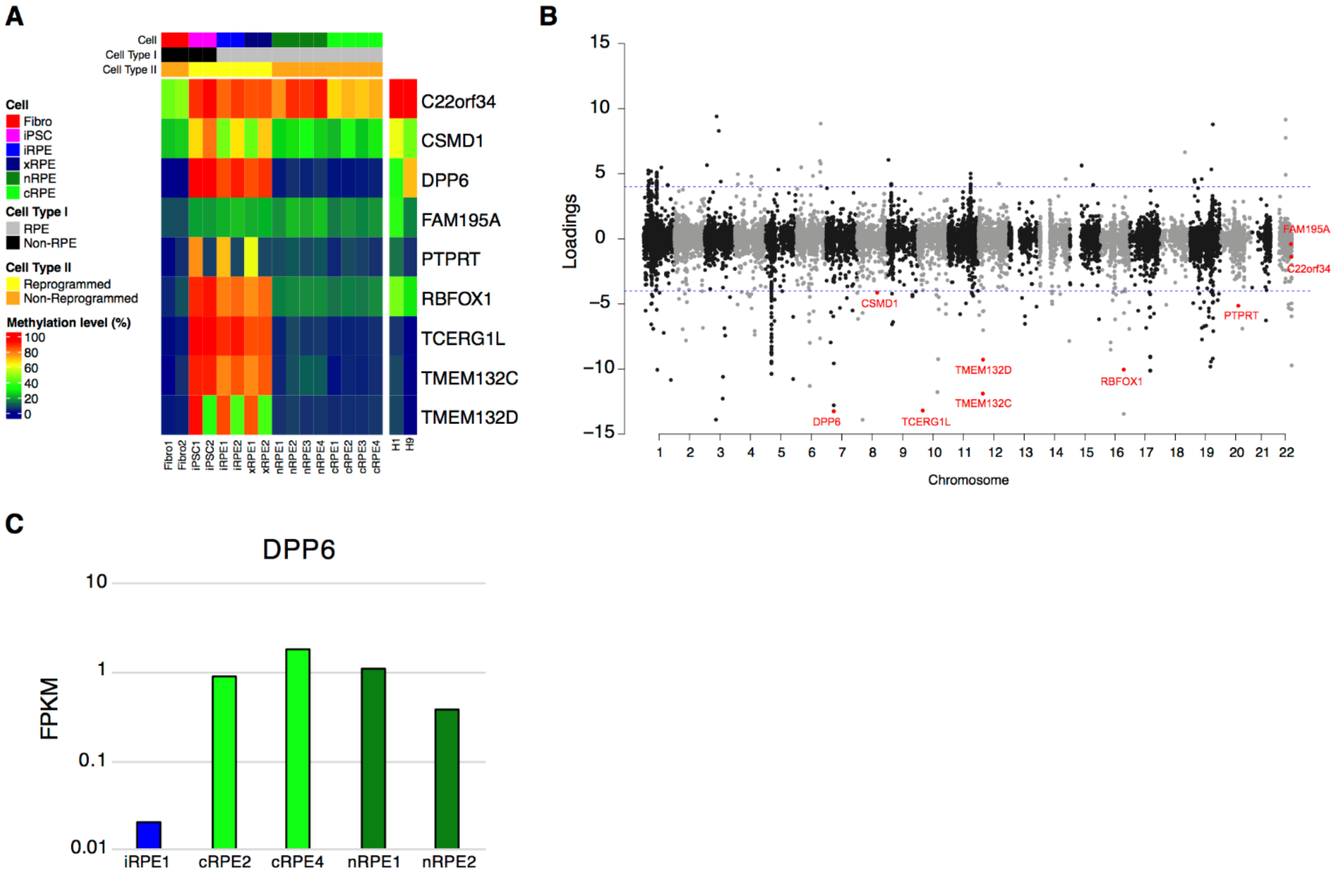

Figure S3. Reprogramming-associated Epigenetic Signatures. Related to Figure 4.

(A) Methylation heat map of reprogramming-associated epigenetic signature genes. Colors indicate the methylation levels of promoters.

(B) Manhattan plot of loadings for IC5 of ICA-based clustering of promoters. Reprogramming-associated epigenetic signature genes are indicated in red. The blue dashed lines indicate the 99th percentiles for the absolute values of loadings.

(C) Expression levels of *DPP6* in iRPE1, cRPE2/4, and nRPE1/2**.**

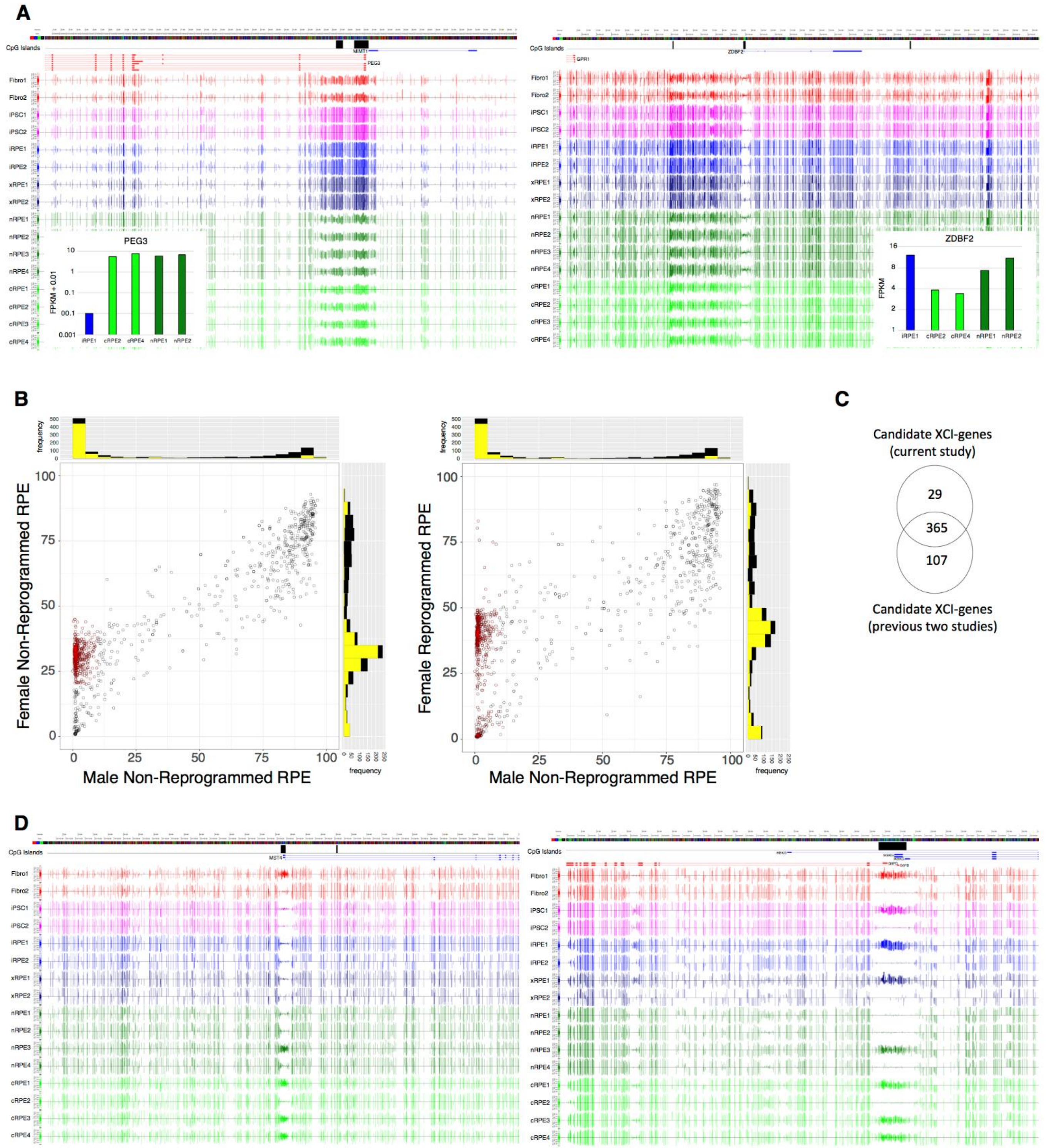

Figure S4. Loss of Imprinting and Erosion of XCI. Related to Figure 4.

(A) Genome browser shots for imprinted genes *PEG3* and *ZDBF2*. From top to bottom, tracks represent CGIs, gene models, and mCG levels of both strands in Fibro1, 2; iPSC1, 2; iRPE1, 2; xRPE1, 2; nRPE1–4; and cRPE1–4. Insets indicate their expression levels.

(B) Identification of candidate XCI-genes. A gene is regarded as a candidate XCI-gene (red circle), if the methylation level of its TSS-proximal region is ≤10% and 20–45% in male and female non-reprogrammed cells, respectively (left panel). Methylation levels of TSS-proximal regions of X-linked genes are also compared between the reprogrammed and non-reprogrammed female RPE cells (right). The yellow portions of the histograms along X- and Y-axes indicate TSS-proximal regions overlapping with CGIs.

(C) Overlap between candidate XCI-genes identified in this study and previous studies (Balaton et al., 2015; Tukiainen et al. 2017).

(D) Genome browser shot for *MST4* and *G6PD*. Methylation patterns of promoter CGIs indicate that XCI appears to be lost and maintained for *MST4* and *G6PD*, respectively.

A

Genes		Down-regulation in iRPE	
		+	–
Proximal hyper-DMRs	+	59	1,195
	–	244	16,543

P value = 3.7e–13, Odds ratio = 3.3

Genes		Up-regulation in iRPE	
		+	–
Proximal hypo-DMRs	+	13	192
	–	131	17,705

P value = 1.0e–08, Odds ratio = 9.1

B

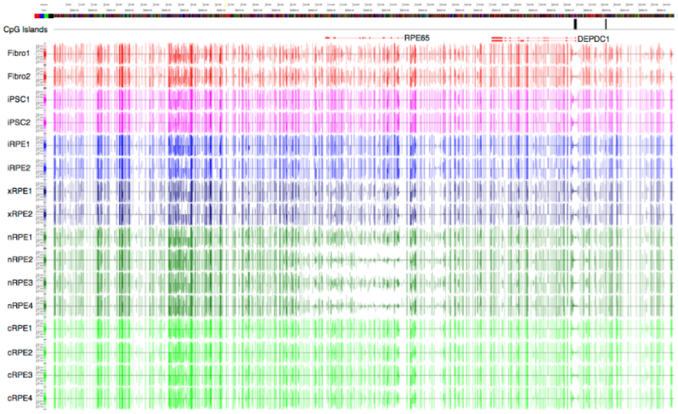

C

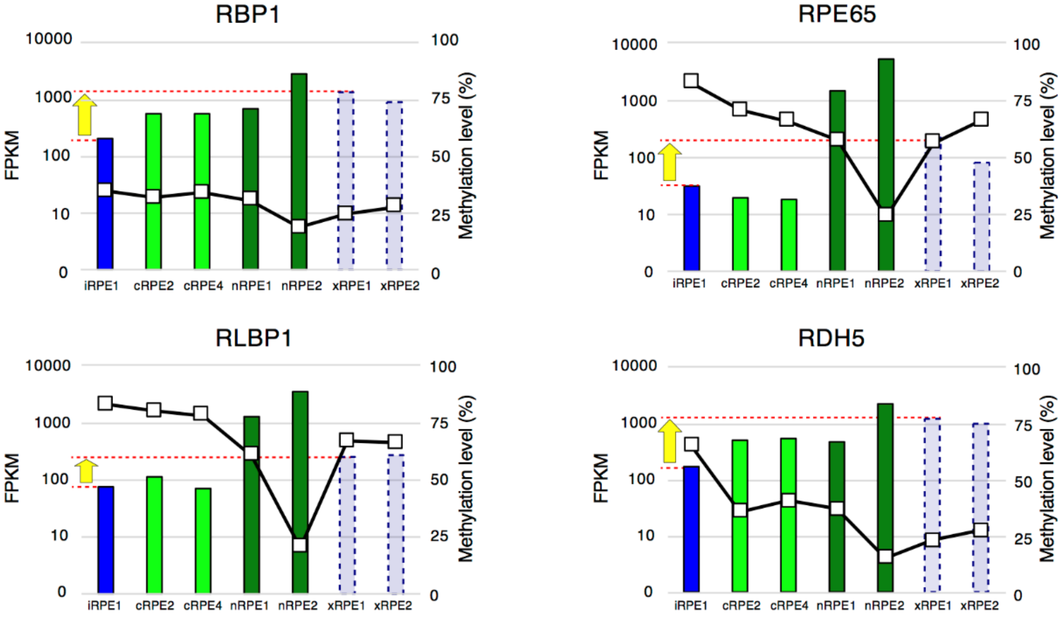

D

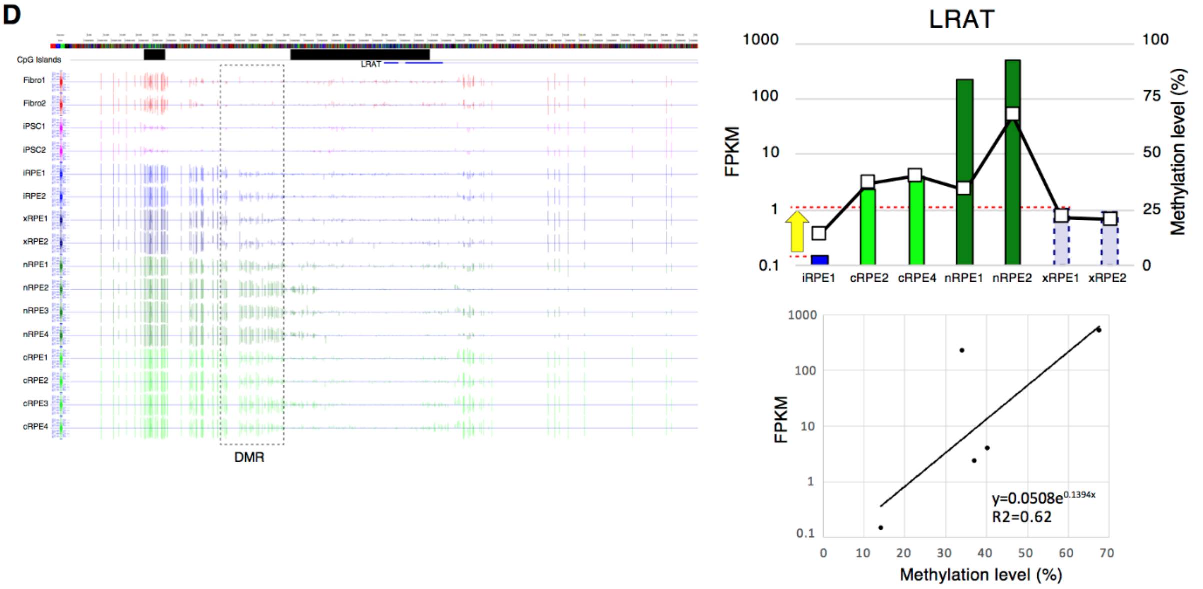

Figure S5. Differential Methylation and Gene Expression. Related to Figure 5.

(A) Correlation between DMRs and DEGs. Co-occurrence is significant between genes proximal to hyper-DMRs and genes down-regulated in iRPE1 (top) and between genes proximal to hypo-DMRs and genes up-regulated in iRPE relative to nRPE (bottom).

(B) Genome browser shot for *RPE65*. From top to bottom, tracks represent CGIs, gene models, and mCG levels of both strands in Fibro1, 2; iPSC1, 2; iRPE1, 2; xRPE1, 2; nRPE1–4; and cRPE1–4. Note that gene body of *RPE65* is hypomethylated in nRPE1–4 compared to the other cells.

(C) Expression levels of 4 visual-cycle genes in xRPE estimated from their gene-body methylation levels. Bars indicate expression levels, and line charts indicate gene-body methylation levels. Bars with dotted lines indicate the estimated expression levels in xRPE. Yellow arrows indicate the estimated levels of induction of individual genes after xenotransplantation.

(D) Differential methylation and expression of *LRAT*. DMR between iRPE and nRPE is bracketed in the genome browser shot (left). Expression levels of *LRAT* in xRPE are estimated from the methylation level of DMR (right top) and the correlation between the methylation level of DMR and expression level of *LRAT* mRNA (right bottom). Yellow arrow indicates the estimated induction of *LRAT* after xenotransplantation.

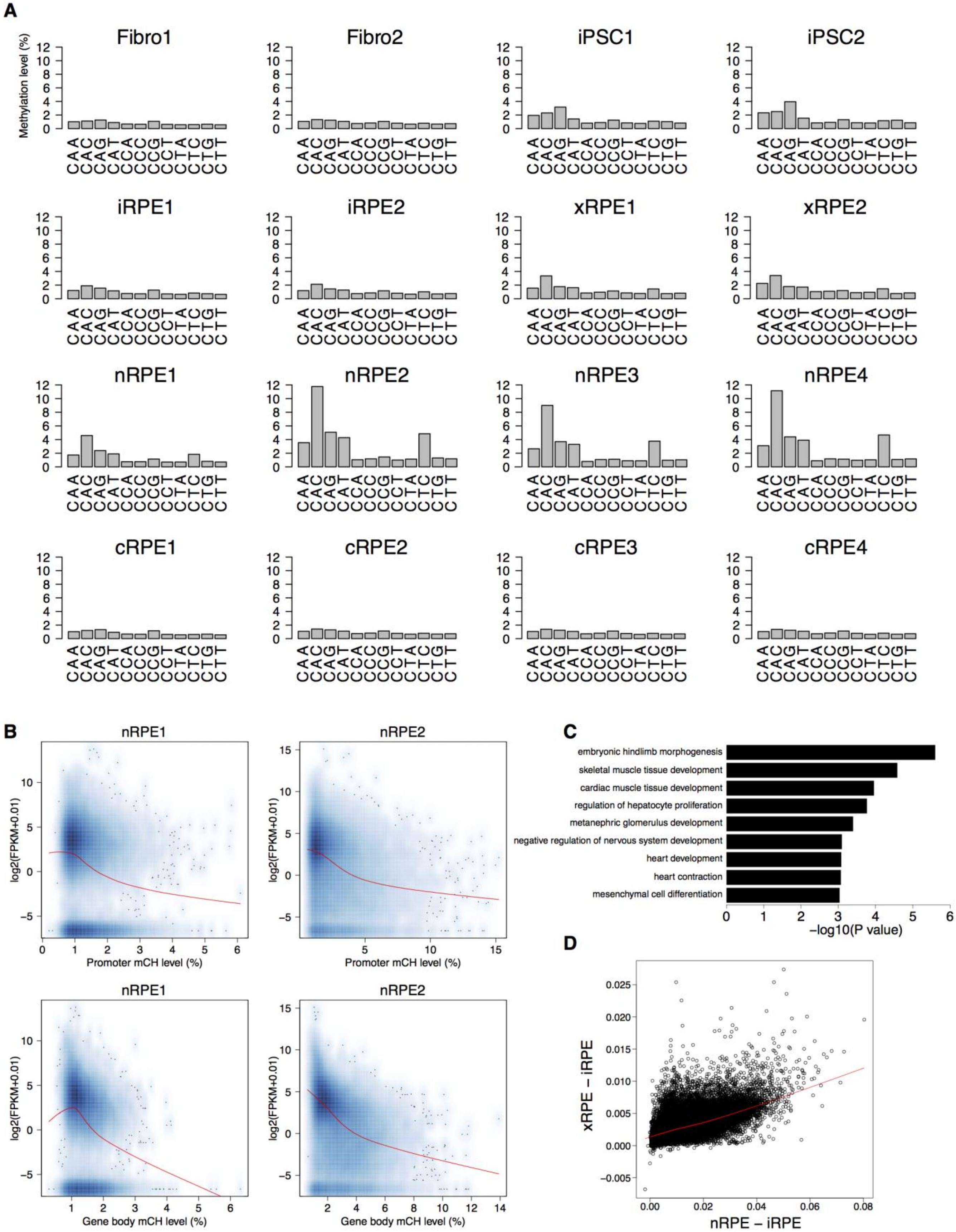

A
Fibro1
Fibro2
iPSC1
iPSC2
iRPE1
iRPE2
xRPE1
xRPE2
nRPE1
nRPE2
nRPE3
nRPE4
cRPE1
cRPE2
cRPE3
cRPE4
Methylation level (%)
CAA CAC CAG CAT CCA CCC CCG CCT CTA CTC CTG CTT
B
nRPE1
nRPE2
log2(FPKM+0.01)
Promoter mCH level (%)
Gene body mCH level (%)
C
embryonic hindlimb morphogenesis
skeletal muscle tissue development
cardiac muscle tissue development
regulation of hepatocyte proliferation
metanephric glomerulus development
negative regulation of nervous system development
heart development
heart contraction
mesenchymal cell differentiation
−log10(P value)
D
xRPE − iRPE
nRPE − iRPE

Figure S6. Characterization of Non-CpG Methylation. Related to Figure 6.

(A) Sequence contexts and mCH levels in each of the 16 methylomes.

(B) Correlation between the levels of mCHs and mRNAs in nRPE1 and nRPE2.

(C) GO enrichment analysis of top 500 genes with high promoter mCH levels in nRPE.

(D) Correlation of mCHs between nRPE and xRPE. Each circle indicates a 100-kb window. For each circle, X- and Y-coordinates indicate the difference of mCH levels (Δ%mCH) between nRPE and iRPE and the Δ%mCH between xRPE and iRPE, respectively.

Supplemental Experimental Procedures

Sample preparation

cRPE: We obtained four distinct batches of normal human retinal pigment cells from Lonza (Lonza, 00194987) and used them as cRPE samples. These cells were cryopreserved, primary cells that were packaged at passage 2. They were guaranteed through 5 population doublings and stain positive (≥ 90%) for pancytokeratin and Zo-1 and negative (≤ 10%) for fibroblast contamination and endothelial marker CD31 (≤ 1%). We spread them on dishes coated with CELLstart (Thermo Fisher). Following cultivation in RtEGM™ BulletKit™ (Lonza, 00195409) supplemented with 2% FBS for 2 to 3 days, we switched the medium to RPE maintenance medium [DMEM low glucose (Sigma-Aldrich, D6046) 350mL, F12 (Sigma-Aldrich, N6658) 150mL, B27 (Thermo Fisher, 17504) 10mL, L-glutamine solution (Sigma-Aldrich, G7513) 5mL, Penicillin-Streptomycin solution (Thermos Fisher, 15140) 5mL] supplemented with 10 ng/mL bFGF (Wako) and 0.5 µM SB431542 (Sigma-Aldrich, S4317).

nRPE: We prepared four nRPE samples from eyes of four healthy donors obtained from SightLife TM (http://www.sightlife.org/, Seattle, WA). We used the SRIRS (simultaneous RPE isolation and RNA stabilization) method based on a unique feature of RNAprotect Cell Reagent (Qiagen 76526) to detaches RPE cells from surgically prepared posterior eye cup, thus providing a highly purified RPE preparation (Wang et al., 2012).

xRPE: We prepared two xRPE samples as described previously (Mandai et al., 2017). Briefly, 1×10^6 iRPE cells were subcutaneously injected with Matrigel in NOG mice. The samples analyzed in this study, xRPE1 and xRPE2, were recovered from the mice at 25 and 9 weeks after transplantation, respectively. Histological examination of these xenografts failed to find any abnormality.

SNP-masked patients' methylome data

We prepared methylation level data on all C's in the reference genome, except for those serving as SNP sites with minor allele frequency > 0.01 in Japanese population as follows. Based on SNP frequency data from TogoVar, an integrated database for Japanese genome variants/variations (https://togovar.biosciencedbc.jp/), we selected 4,558,776 C's from both strands of the reference genome (i.e., 0.39% of total C's) and masked them from the methylation level data for the prevention of potential personal identification. The data include the numbers of reads supporting methylated and unmethylated status of each C.

RNA-seq

RNA-Seq libraries were prepared and sequenced as described previously (Mandai et al., 2017). RNA-Seq reads were mapped to the hg19 reference genome using Tophat2. Cufflinks (Trapnell et al., 2010) was used to generate the normalized expression levels of genes expressed as FPKM values.

Mapped reads were assigned to all exons using featureCounts (Liao et al., 2014). To filter out genes with low expression levels, we considered only those with at least 10 read counts in either iRPE1 or nRPE1/2. The differential analysis was conducted using the Bioconductor package edgeR (Robinson et al., 2009), applying TMM (trimmed Mean of M-values) library normalization. A gene was defined as differentially expressed, if the false discovery rate (FDR)

corrected with Benjamini-Hochberg method was less than 0.01 and if the log2 fold change (log2FC) was more than 1 (up-regulated) or less than −1 (down-regulated).

Software and algorithm

Versions and sources of the softwares/algorithms used in this study are summarized in the following table.

REAGENT or RESOURCE	IDENTIFIER	SOURCE
ComplexHeatmap (v1.12.0)	Gu et al., 2016	https://bioconductor.org/packages/release/bioc/html/ComplexHeatmap.html
edgeR (v3.16.5)	Robinson et al., 2010	https://bioconductor.org/packages/release/bioc/html/edgeR.html
fastICA (v1.2-1)	Marchini et al., 2017	https://cran.r-project.org/web/packages/fastICA/index.html
featureCounts (v1.4.6)	Liao et al., 2014	http://bioinf.wehi.edu.au/featureCounts/
HOMER (v4.9.1)	Heinz et al., 2010	http://homer.ucsd.edu/homer/index.html
metilene (v0.2-6)	Jühling et al., 2016	https://www.bioinf.uni-leipzig.de/Software/metilene/
R (v3.3.3)	R Core Team	http://www.R-project.org/
topGO (v2.26.0)	Alexa and Rahnenfuhrer, 2016	http://bioconductor.org/packages/release/bioc/html/topGO.html

Supplemental References

Balaton, B.P., Cotton, A.M., and Brown, C.J. (2015). Derivation of consensus inactivation status for X-linked genes from genome-wide studies. Biol. Sex Differ. 6, 35.

Gu, Z., Eils, R., and Schlesner, M. (2016). Complex heatmaps reveal patterns and correlations in multidimensional genomic data. Bioinformatics 32, 2847–2849.

Heinz, S., Benner, C., Spann, N., Bertolino, E., Lin, Y.C., Laslo, P., Cheng, J.X., Murre, C., Singh, H., and Glass, C.K. (2010). Simple combinations of lineage-determining transcription factors prime cis-regulatory elements required for macrophage and B cell identities. Mol. Cell 38, 576–589.

Jühling, F., Kretzmer, H., Bernhart, S.H., Otto, C., Stadler, P.F., and Hoffmann, S. (2016). metilene: fast and sensitive calling of differentially methylated regions from bisulfite sequencing data. Genome Res. 26, 256–262.

Liao, Y., Smyth, G.K., and Shi, W. (2014). featureCounts: an efficient general purpose program for assigning sequence reads to genomic features. Bioinformatics 30, 923–930.

Mandai, M., Watanabe, A., Kurimoto, Y., Hirami, Y., Morinaga, C., Daimon, T., Fujihara, M., Akimaru, H., Sakai, N., Shibata, Y., et al. (2017). Autologous induced stem-cell-derived retinal cells for macular degeneration. N. Engl. J. Med. 376, 1038–1046.

Robinson, M.D., McCarthy, D.J., and Smyth, G.K. (2010). edgeR: a Bioconductor package for differential expression analysis of digital gene expression data. Bioinformatics 26, 139–140.

Trapnell, C., Williams, B.A., Pertea, G., Mortazavi, A., Kwan, G., van Baren, M.J., Salzberg, S.L., Wold, B.J., and Pachter, L. (2010). Transcript assembly and quantification by RNA-seq reveals unannotated transcripts and isoform switching during cell differentiation. Nat. Biotechnol. 28, 511–515.

Tukiainen, T., Villani, A.C., Yen, A., Rivas, M.A., Marshall, J.L., Satija, R., Aguirre, M., Gauthier, L., Fleharty, M., Kirby, A. et al. (2017). Landscape of X chromosome inactivation across human tissues. Nature 550, 244–248.

Wang, C.X-Z., Zhang, K., Aredo, B., Lu, H., and Ufret-Vincenty, R.L. (2012) Novel method for the rapid isolation of RPE cells specifically for RNA extraction and analysis. Exp. Eye Res. 102, 1–9.
